# Post-transcriptional repression of mRNA enhances competence to transit from mitosis to meiosis in mouse spermatogenic cells

**DOI:** 10.1101/2023.09.20.557439

**Published:** 2023-09-22

**Authors:** Maria M. Mikedis, Bingrun Liu, Dirk G. de Rooij, David C. Page

**Affiliations:** a Reproductive Sciences Center, Division of Developmental Biology, Cincinnati Children’s Hospital Medical Center, Cincinnati, OH, United States; b Department of Pediatrics, University of Cincinnati College of Medicine, Cincinnati, OH United States; c Whitehead Institute, Cambridge, MA, United States; d Department of Biology, Massachusetts Institute of Technology, Cambridge, MA, United States; e Howard Hughes Medical Institute, Whitehead Institute, Cambridge, MA, United States

## Abstract

The special cell cycle known as meiosis transforms diploid germ cells into haploid gametes. In mammalian testes, diploid spermatogenic cells become competent to transition from mitosis to meiosis in response to retinoic acid. In mice, previous studies revealed that MEIOC, alongside binding partners YTHDC2 and RBM46, represses mitotic genes and promotes robust meiotic gene expression in spermatogenic cells that have already initiated meiosis. Here, we molecularly dissect MEIOC-dependent regulation in mouse spermatogenic cells and find that MEIOC actually shapes the transcriptome much earlier, even before meiotic initiation. MEIOC, acting with YTHDC2 and RBM46, destabilizes mRNA targets, including transcriptional repressors *E2f6* and *Mga*, in mitotic spermatogonia. MEIOC thereby derepresses E2F6- and MGA-repressed genes, including *Meiosin* and other meiosis-associated genes. This confers on spermatogenic cells the molecular competence to, in response to retinoic acid, fully activate the STRA8-MEIOSIN transcriptional regulator, which is required for the meiotic G1/S cell cycle transition and meiotic gene expression. We conclude that in mice, mRNA decay mediated by MEIOC-YTHDC2-RBM46 enhances the competence of mitotic spermatogonia to transit from mitosis to meiosis.

## INTRODUCTION

Sexual reproduction depends on meiosis – a specialized cell cycle that produces haploid gametes via one round of DNA replication followed by two rounds of chromosome segregation. The major chromosomal events of meiosis, including pairing, synapsis, and crossing over of homologous chromosomes, are generally conserved across eukaryotes. By contrast, the mechanisms that govern the transition from mitosis to meiosis are less well conserved (Kimble 2011). For example, in budding yeast, meiotic initiation occurs when multiple inputs converge to activate a master transcription factor (IME1) that upregulates meiotic gene expression (reviewed in van Werven and Amon 2011; Kassir et al. 1988; Smith et al. 1990). In *Drosophila melanogaster*, the transition from mitosis to meiosis occurs via translational repression mediated by the RNA helicase Bgcn and its binding partner Bam (Gonczy et al. 1997; McKearin and Spradling 1990; Chen et al. 2014; Insco et al. 2009, 2012).

In mammals, transcriptional activation induced by extrinsic signaling plays a central role in the mitosis-to-meiosis (i.e., meiotic G1/S) transition. Retinoic acid induces meiotic entry by activating the gene expression of *Stra8* and *Meiosin* (Baltus et al. 2006; Anderson et al. 2008a; Dokshin et al. 2013; Ishiguro et al. 2020). Acting as a heterodimer, STRA8-MEIOSIN transcriptionally activates the robust expression of G1/S cyclins as well as meiotic factors that orchestrate the chromosomal events of meiotic prophase I (Baltus et al. 2006; Anderson et al. 2008a; Soh et al. 2015; Kojima et al. 2019; Ishiguro et al. 2020; Mark et al. 2008). In mice on a C57BL/6 genetic background, premeiotic oocytes and preleptotene spermatocytes with *Stra8* or *Meiosin* genetically ablated halt development and fail to progress into meiotic prophase I (Anderson et al. 2008a; Baltus et al. 2006; Dokshin et al. 2013; Ishiguro et al. 2020). In addition, *Stra8*-null premeiotic oocytes and preleptotene spermatocytes fail to undergo meiotic DNA replication (Anderson et al. 2008a; Baltus et al. 2006; Dokshin et al. 2013).

In the mammalian testis, competence to transit from mitosis to meiosis in response to retinoic acid is acquired during spermatogenesis. In mitotic spermatogonia, DMRT1 postpones the acquisition of competence, thereby preventing precocious meiotic entry, by repressing retinoic acid-dependent transcription as well as *Stra8* and likely *Meiosin* gene expression (Matson et al. 2010; Ishiguro et al. 2020). As a result, undifferentiated spermatogonia exposed to endogenous retinoic acid express low levels *Stra8*, but do not express *Meiosin*, and consequently become mitotic differentiating spermatogonia (Matson et al. 2010; Ishiguro et al. 2020). Differentiating spermatogonia prematurely exposed to exogenous retinoic acid may express STRA8 protein, but they do not initiate meiosis (Johnson et al. 2023; Endo et al. 2015). At the mitosis-to-meiosis transition, the SCF E3 ubiquitin ligase complex degrades DMRT1 (Matson et al. 2010; Nakagawa et al. 2017), thereby conferring upon spermatogenic cells the competence to express *Stra8* and *Meiosin* and initiate meiosis in response to retinoic acid. To date, the SCF complex is the only known positive regulator of the spermatogenic cells’ competence to transit from mitosis to meiosis in response to retinoic acid.

Post-transcriptional regulation of mRNA plays a role in governing early meiotic prophase I in mammals. MEIOC acts together with RNA helicase YTHDC2 and RNA-binding protein RBM46 to regulate entry into and progression through meiotic prophase I (Abby et al. 2016; Soh et al. 2017; Wojtas et al. 2017; Bailey et al. 2017; Hsu et al. 2017; Jain et al. 2018; Qian et al. 2022). During meiotic prophase I, MEIOC is required to increase meiotic gene expression and repress the mitotic cell cycle program, with the latter of these two functions inhibiting a premature and aberrant metaphase several days before wild-type meiotic metaphase I. Consistent with MEIOC’s distant homology to Bam and YTHDC2’s homology to Bgcn, the MEIOC-YTHDC2-RBM46 complex binds to mRNA (Hsu et al. 2017; Soh et al. 2017; Abby et al. 2016; Bailey et al. 2017; Li et al. 2022; Saito et al. 2022; Jain et al. 2018). YTHDC2 interacts with exoribonuclease XRN1, while RBM46 recruits UPF1, a mediator of nonsense mediated decay, and subunits of the cytoplasmic deadenylase CCR4-NOT complex, which suggests that YTHDC2 and RBM46’s mRNA targets are degraded (Wojtas et al. 2017; Li et al. 2022; Qian et al. 2022; Kretschmer et al. 2018). However, how the MEIOC-YTHDC2-RBM46 complex post-transcriptionally regulates its mRNA targets during entry into meiotic prophase I remains poorly defined.

Here we molecularly dissect MEIOC’s activity in mouse spermatogenic cells during the mitosis-to-meiosis transition via two parallel approaches: (i) single-cell RNA sequencing (scRNA-seq) analysis of postnatal testes and (ii) bulk RNA-seq analysis of developmentally synchronized testes with histologically verified staging. Both approaches reveal that *Meioc*-null germ cells developmentally diverge from wild-type spermatogenic cells at the meiotic G1/S transition, earlier than previously appreciated. Furthermore, we find that MEIOC’s activity leads to an increase in meiosis-associated gene expression in late mitotic spermatogonia, before meiotic initiation. We discover that MEIOC destabilizes mRNAs encoding transcriptional repressors of meiotic gene expression. This repression of repressors facilitates activation of the master meiotic transcriptional regulator STRA8-MEIOSIN in response to retinoic acid. Therefore, a post-transcriptional repressor of mRNA, acting in parallel to the SCF complex’s degradation of DMRT1, enhances spermatogenic cells’ competence to activate the meiotic transcriptional regulator and transition from mitosis to meiosis.

## RESULTS

### MEIOC’s activity facilitates a major transcriptomic shift at the meiotic G1/S transition

To characterize the molecular consequences of MEIOC activity on spermatogenesis, we performed 10x Genomics Chromium-based scRNA-seq on *Meioc*-null and wild-type P15 testes and identified germ cell clusters by cell type-enriched marker expression and transcriptome-based cell cycle analysis (Fig. S1–3; Supplemental Information). These germ cell clusters consisted of four clusters in mitosis (undifferentiated spermatogonia [Undiff], differentiating types A_1_-A_4_ spermatogonia [A1–4], differentiating Intermediate and type B spermatogonia [In/B], and differentiating type B spermatogonia in G2/M phase [B G2M]); three clusters spanning the mitosis-to-meiosis transition (preleptotene spermatocytes in G1, early S, and late S phase [pL G1, pL eS, and pL lS, respectively]); and three clusters in meiotic prophase I (leptotene spermatocytes [L], zygotene spermatocytes [Z], and pachytene spermatocytes [P]).

**Figure 1:**
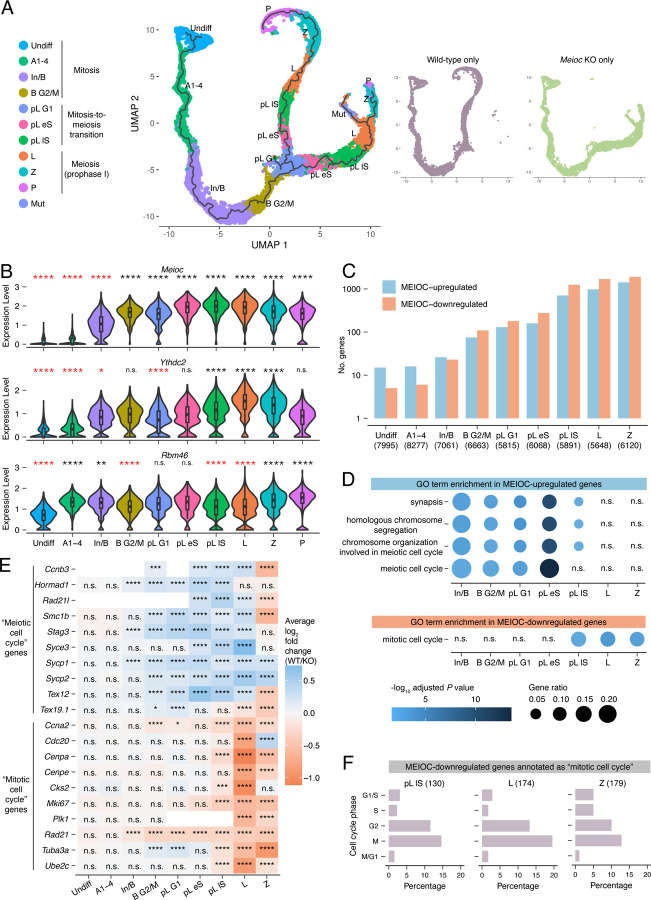
*Meioc*-null germ cells transcriptomically diverge from wild-type germ cells during the G1/S phase transition. A: UMAP visualization with pseudotime trajectory of wild-type and *Meioc* KO germ cells from P15 testes. Large plot on left displays both wild-type and *Meioc* KO cells. Smaller plots on right display cells of a single genotype. B: Expression levels of *Meioc*, *Ythdc2*, and *Rbm46* in germ cell clusters from wild-type testes. Black and red asterisks designate enrichment or depletion, respectively, relative to all other germ cells. Clusters marked as “not done” (n.d.) did not meet expression thresholds set for statistical testing. C: Number of genes identified as MEIOC-upregulated (log_2_ fold change (WT/KO)>0.1, adj. *P*<0.05) and -downregulated (log_2_ fold change (WT/KO)<-0.1, adj. *P*<0.05) within each germ cell cluster. D: Gene Ontology (GO) term enrichment analysis for MEIOC-upregulated and -downregulated genes shown. Graph for MEIOC-upregulated genes displays top 4 enriched categories identified in the B G2/M cluster. Graph for MEIOC-downregulated genes displays a selected category of interest. E: Heatmap of log_2_ fold changes for selected genes annotated as GO terms “meiotic cell cycle” or “mitotic cell cycle”. F: Associated cell cycle phases for MEIOC-downregulated genes that are annotated as GO term “mitotic cell cycle.” Only those genes whose expression is associated with a specific cell cycle phase are graphed. Cluster abbreviations: Undiff, undifferentiated spermatogonia; A1–4, differentiating types A_1_-A_4_ spermatogonia; In/B, differentiating Intermediate and type B spermatogonia; B G2M, differentiating type B spermatogonia in G2/M phase; pL G1, preleptotene spermatocytes in G1 phase; pL eS, preleptotene spermatocytes in early S; pL lS, preleptotene spermatocytes in late S phase; L, leptotene spermatocytes; Z, zygotene spermatocytes; P, pachytene spermatocytes; Mut, mutant spermatocytes. *, adj. *P*<0.05; **, adj. *P*<0.01; ***, adj. *P*<0.001; ****, adj. *P*<0.0001; n.s., not significant.

We then applied pseudotime analysis to reconstruct the germ cells’ developmental trajectory from undifferentiated spermatogonia to pachytene spermatocytes (Fig. 1A). *Meioc*-null germ cells followed the same trajectory as wild-type cells during the mitotic stages of spermatogenesis but diverged in the G1 phase of the preleptotene stage, just before meiotic S phase begins. We used bulk RNA-seq analysis of testes developmentally synchronized and histologically verified for preleptotene spermatocytes to confirm that MEIOC is associated with transcriptomic changes at this stage, including increased abundance of G1/S transcripts (Fig. S4A–D; Supplemental Information). This demonstrates that MEIOC drives a major transcriptomic shift in spermatogenic cells at the meiotic G1/S transition.

### MEIOC predominantly affects the abundance of meiotic, rather than mitotic, transcripts before the meiotic G1/S transition

MEIOC protein is first detected immunohistochemically at the preleptotene stage (Soh et al. 2017; Abby et al. 2016). We found that *Meioc* transcripts became highly abundant at an earlier stage, in late mitotic spermatogonia (In/B and B G2M clusters) (Fig. 1B). Binding partner *Ythdc2* followed a similar pattern of expression, while *Rbm46* was highly abundant in all germ cell clusters examined. These observations raised the possibility that MEIOC, potentially in collaboration with YTHDC2 and RBM46, shapes the germline transcriptome in late mitotic spermatogonia, before the protein can be reliably detected via immunostaining.

To assess how MEIOC impacts the transcriptome during spermatogenesis, we carried out scRNA-seq-based differential expression analysis in germ cell clusters associated with mitotic spermatogonia (Undiff, A1–4, In/B, and B G2M clusters), the mitosis-to-meiosis transition (pL G1, eS, and lS clusters), and meiotic prophase I (L and Z clusters), and identified genes whose transcript abundance is upregulated (log_2_ fold change WT/*Meioc* KO > 0.1 and adjusted *P* < 0.05) or downregulated (log_2_ fold change WT/*Meioc* KO < −0.1 and adjusted *P* < 0.05) (Fig. 1C) by MEIOC. The number of differentially abundant transcripts increased in the In/B and B G2/M clusters, mirroring *Meioc* and *Ythdc2* expression patterns, and continued to expand thereafter. We conclude that MEIOC affects the germline transcriptome before the meiotic G1/S transition.

Based on functional analysis, transcripts upregulated by MEIOC in the In/B through L clusters were enriched for annotations related to meiosis (Fig. 1D). Several transcripts contributing to this enrichment became more abundant in mitotic spermatogonia and remained more abundant in early meiotic spermatocytes (Fig. 1E). Bulk RNA-seq analysis of preleptotene-enriched testes also revealed enrichment of meiosis-associated factors among MEIOC-upregulated transcripts (Fig. S4C, E). Therefore, MEIOC’s activity leads to increased meiotic transcript abundance in late mitotic spermatogonia, earlier than previously appreciated.

Given that MEIOC decreases mitotic transcript abundance in P10 testes (Soh et al. 2017), we asked whether these transcriptomic changes were also induced by MEIOC in mitotic spermatogonia. MEIOC-downregulated transcripts were enriched in “mitotic cell cycle” factors in the pL lS to Z clusters but not in developmentally earlier clusters (Fig. 1D,E). Furthermore, based on a curated list of genes whose expression is linked to specific cell cycle phases (Hsiao et al. 2020), these MEIOC-downregulated “mitotic cell cycle” genes were primarily associated with G2 or M phases (Fig. 1F). Therefore, the downregulation of “mitotic cell cycle” transcripts occurs primarily after the meiotic G1/S transition and is consistent with MEIOC preventing an aberrant M phase during meiotic prophase I.

In sum, MEIOC increases the abundance of meiosis-associated transcripts beginning in late transit-amplifying spermatogonia. Only later in the mitosis-to-meiosis transition does MEIOC decrease mitotic transcript abundance.

### MEIOC destabilizes its target transcripts

**Figure 2:**
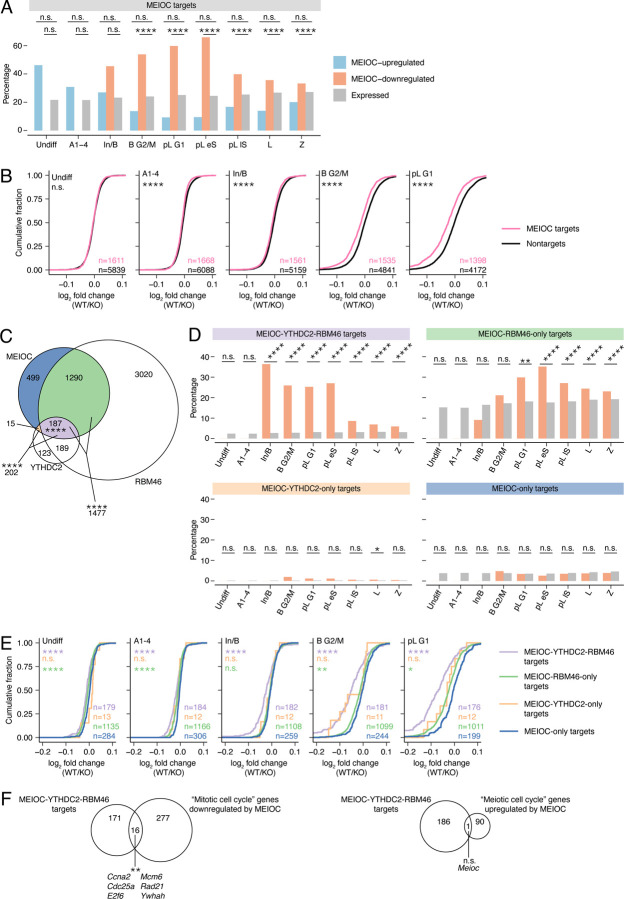
MEIOC-YTHDC2-RBM46 destabilize their mRNA targets. A: Percentage of MEIOC targets within MEIOC-upregulated, MEIOC-downregulated, and expressed genes, defined by scRNA-seq. MEIOC targets were identified from reanalysis of a published RIP-seq dataset (Soh et al., 2017). B: Cumulative fraction for log_2_ fold change (WT / *Meioc* KO), defined by scRNA-seq, with genes binned as MEIOC targets or nontargets. C: Overlap of mRNAs identified by MEIOC RIP-seq, and YTHDC2 CLIP-seq, and RBM46 eCLIP-seq datasets. MEIOC RIP-seq data were reanalyzed from Soh et al., 2017. YTHDC2 CLIP-seq analysis was published in Saito et al., 2022. RBM46 eCLIP-seq data were reanalyzed from Qian et al., 2022. Asterisks denote statistical enrichment. D: Percentage of MEIOC-YTHDC2-RBM46 targets, MEIOC-RBM46-only targets, MEIOC-YTHDC2-only targets, and MEIOC-only targets within MEIOC-downregulated and expressed genes. E: Cumulative fraction for log_2_ fold change (WT / *Meioc* KO), defined by scRNA-seq, with genes binned as MEIOC-YTHDC2-RBM46 targets, MEIOC-RBM46-only targets, MEIOC-YTHDC2-only targets, and MEIOC-only targets. Asterisks represent comparison of color-matched target set to MEIOC-only targets. F: MEIOC-YTHDC2-RBM46 targets are enriched for GO term “mitotic cell cycle” genes downregulated by MEIOC but not GO term “meiotic cell cycle” genes upregulated by MEIOC. Upregulated and downregulated genes were defined by scRNA-seq (any germ cell cluster). Asterisks represent statistical enrichment; “n.s.” represents “not significant” statistical enrichment or depletion. *, adj. *P*<0.05; **, adj. *P*<0.01; ***, adj. *P*<0.0001; ****, adj. *P*<0.0001; n.s., not significant.

In early spermatocytes, MEIOC localizes to the cytoplasm and interacts with RNA-binding proteins YTHDC2 and RBM46, which recruit additional proteins that degrade mRNA (Wojtas et al. 2017; Li et al. 2022; Qian et al. 2022; Kretschmer et al. 2018). Therefore, we hypothesized that MEIOC degrades its mRNA targets. We first defined transcripts that associate with MEIOC by reanalyzing our previously published MEIOC RIP-seq data from P15 testes (Soh et al. 2017), identifying 1,991 MEIOC-bound mRNA targets. We then assessed the molecular impact of MEIOC’s interaction with these transcripts via two approaches. First, we examined these targets’ representation among transcripts exhibiting statistically significant MEIOC-dependent changes in abundance. We found that MEIOC targets were enriched among transcripts whose abundance decreased, but not increased, in response to MEIOC, in the B G2/M through Z clusters (Fig. 2A). Second, we examined all MEIOC targets as a group, irrespective of whether they met a statistical cut off in the differential expression analysis. MEIOC targets exhibited slightly lower fold changes (WT/KO) than nontargets in the A1–4 through Z clusters (Fig. 2B). A parallel analysis of computationally estimated changes in transcript stability from bulk RNA-seq data showed that MEIOC targets had reduced transcript stability relative to nontargets (Fig. S6A). Therefore, MEIOC decreases the abundance of its target transcripts, presumably by promoting post-transcriptional degradation, beginning in mitotic spermatogonia, before the mitosis-to-meiosis transition.

Given MEIOC’s interaction with YTHDC2 and RBM46, we hypothesized that many MEIOC targets are also bound by YTHDC2 and RBM46. Based on recently published YTHDC2 and RBM46 CLIP data from the testis (Saito et al. 2022; Qian et al. 2022), MEIOC shared 202 mRNA targets with YTHDC2 and 1,477 targets with RBM46, both of which are statistically significant overlaps (Fig. 2C). All three proteins shared 187 targets. Only 15 mRNAs were bound by MEIOC and YTHDC2 but not RBM46; this small group likely represents technical differences between datasets rather than a biologically meaningful set of transcripts. We conclude that MEIOC shares many, but not all, of its mRNA targets with YTHDC2 and RBM46.

As YTHDC2 and RBM46 interact with proteins that degrade mRNA, we hypothesized that MEIOC’s interaction with YTHDC2 and RBM46 has a greater impact on transcript stability than MEIOC alone. We subsequently binned MEIOC’s targets as: MEIOC-RBM46-YTHDC2 targets, MEIOC-RBM46-only targets, MEIOC-YTHDC2-only, and MEIOC-only targets. We then compared how these groups respond to MEIOC in our scRNA-seq data. First, we examined whether each of these groups was overrepresented among transcripts whose abundance decreased in response to MEIOC. MEIOC-RBM46-YTHDC2 targets exhibited enrichment beginning in mitotic spermatogonia (Fig. 2D). By contrast, MEIOC-RBM46-only targets were enriched beginning at the transition from mitosis to meiosis, while MEIOC-only targets showed no enrichment (Fig. 2D). Second, we examined the effect of YTHDC2 and RBM46 on the stability of all MEIOC targets, irrespective of statistical cutoff. Relative to MEIOC-only targets, MEIOC-RBM46-YTHDC2 targets exhibited the largest decrease in transcript abundance in all scRNA-seq germ cell clusters (Fig. 2E), as well as in transcript stability in the bulk RNA-seq analysis of preleptotene-enriched testes (Fig. S5B, S6B). Taken together, these data demonstrate that MEIOC’s destabilization of its target mRNAs occurs via its interaction with both YTHDC2 and RBM46.

Given that MEIOC-downregulated transcripts are enriched for “mitotic cell cycle” factors, we hypothesized that MEIOC-YTHDC2-RBM46 is directly targeting and destabilizing these factors. By comparing MEIOC-downregulated “mitotic cell cycle” transcripts to MEIOC-YTHDC2-RBM46 targets, we discovered a small but statistically significant overlap that included factors that impact cell cycle progression, such as *Ccna2* and *E2f6* (Fig. 2F). Some targets were downregulated by MEIOC in mitotic spermatogonia and early during the mitosis-to-meiosis transition, before “mitotic cell cycle” enrichment was evident among the MEIOC-downregulated genes (Fig. 1C,D). Therefore, MEIOC’s destabilization of mRNAs before and during the mitosis-to-meiosis transition may impact spermatogenic cells’ ability to establish a meiosis-specific cell cycle program in meiotic prophase I spermatocytes.

We also considered the possibility that not all MEIOC-YTHDC2-RBM46 targets are similarly regulated. In particular, given that MEIOC-upregulated transcripts are enriched for “meiotic cell cycle” factors (Fig. 1C,D), we asked whether these transcripts were MEIOC-YTHDC2-RBM46 targets. MEIOC-upregulated “meiosis cell cycle” transcripts and MEIOC-YTHDC2-RBM46 targets shared one transcript, *Meioc* (Fig. 2F), with this overlap representing neither statistical depletion nor enrichment. Therefore, MEIOC-YTHDC2-RBM46 does not directly regulate the stability of meiosis-associated transcripts.

In total, MEIOC promotes the decay of its target transcripts beginning in mitotic spermatogonia by acting with YTHDC2 and RBM46.

### MEIOC’s destabilization of *E2f6* and *Mga* mRNA increases expression of E2F6- and MGA-repressed genes

**Figure 3:**
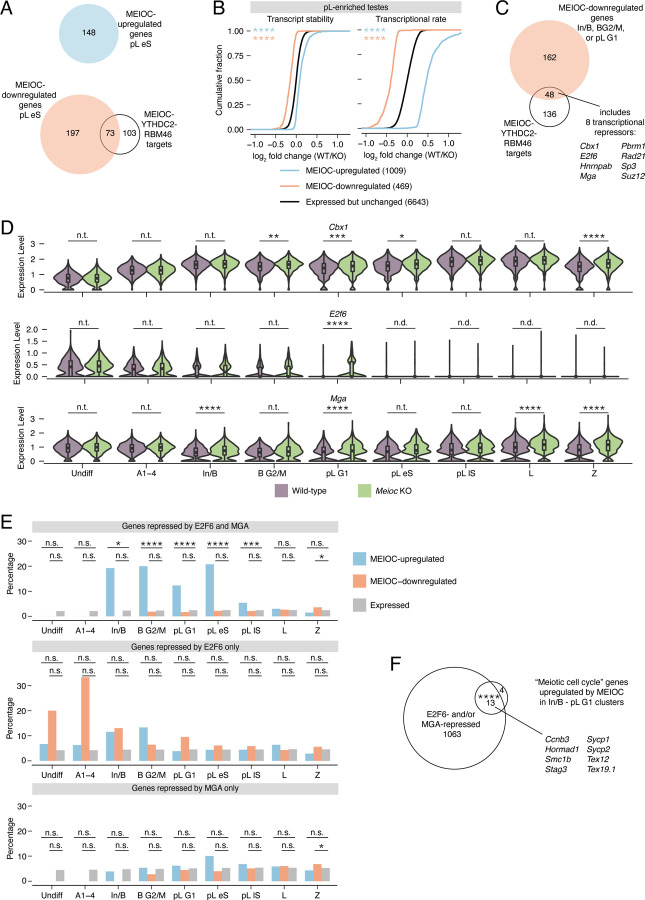
MEIOC-YTHDC2-RBM46’s repression of *E2f6* and *Mga* mRNA relieves E2F6- and MGA-mediated transcriptional repression. A: Venn diagram comparing MEIOC-upregulated and -downregulated genes in pL eS cluster to MEIOC-YTHDC2-RBM46 targets. The majority of genes whose transcript abundance changes in response to MEIOC are not directly targeted by MEIOC-YTHDC2-RBM46. B: Cumulative distribution of log_2_ fold change (WT/ *Meioc* KO) for transcript stability (left) and transcriptional rate (right) for MEIOC-upregulated and MEIOC-downregulated genes compared to genes that expressed but do not change in response to MEIOC. Transcript stabilities and transcriptional rates were estimated using bulk RNA-seq data from preleptotene-enriched testes. Asterisks represent comparison of color-matched gene set to expressed but not regulated gene set. C: Identification of MEIOC-YTHDC2-RBM46 targets that are downregulated by MEIOC in In/B, B G2/M, and/or pL G1 clusters. Of this set, 8 mRNAs encode proteins that have been reported to function as transcriptional repressors. D: Expression levels for PRC1.6 subunits *Cbx1*, *E2f6*, and *Mga* in wildtype vs. *Meioc*-null cells in all germ cell clusters identified. *Cbx1*, *E2f6*, and *Mga* are downregulated by MEIOC in the In/B, B G2/M, and/or pL G1 clusters (adj. P < 0.05). E: Percentage of genes repressed by E2F6 and MGA (top), E2F6 only (center), and MGA only (bottom) among MEIOC-upregulated, MEIOC-downregulated, and expressed genes. E2F6-repressed genes and MGA-repressed genes were identified via reanalysis of published ChIP-seq and RNA-seq datasets from mouse embryonic stem cells (Dahlet et al., 2021; Stielow et al., 2018; Zin et al., 2021). F: Overlap between E2F6- and/or MGA-repressed genes and the “meiotic cell cycle” genes upregulated by MEIOC in In/B, B G2/M, and pL G1 clusters. Example genes that fall within this overlap are listed. *, adj. *P*<0.05; **, adj. *P*<0.01; ***, adj. *P*<0.001; ****, adj. *P*<0.0001; n.s., not significant; n.t., not tested (comparison was excluded from statistical testing because log_2_ fold change> −0.1 and <0.1); n.d., not detected (transcript expressed in <25% cells in each population being compared).

The majority of MEIOC-regulated transcripts, as defined by RNA-seq analysis of wild-type versus *Meioc*-null samples, do not directly interact with MEIOC-YTHDC2-RBM46 (Fig. 3A, S6C). We examined whether these changes were dominated by altered transcript stabilities or transcriptional rates. By applying REMBRANDTS to our bulk RNA-seq data, we estimated change in mRNA abundance as change in exonic reads, change in pre-mRNA abundance (i.e., transcriptional rate) as change in intronic reads, and change in mRNA stability as the difference between the change in exonic reads and that in intronic reads (Alkallas et al. 2017; Gaidatzis et al. 2015). MEIOC-regulated transcripts exhibited large changes in transcriptional rates and smaller changes in transcript stabilities (Fig. 3B). Therefore, MEIOC is mediating changes in transcription, which then changes the abundance of transcripts not bound by MEIOC.

Next, we sought to identify MEIOC targets that drive transcriptional changes. The vast majority of MEIOC-upregulated transcripts are not directly bound by MEIOC (Fig. 2A, 3A). We hypothesized that MEIOC-YTHDC2-RBM46 destabilizes a mRNA that encodes a transcriptional repressor. Destabilization of this mRNA consequently derepresses (i.e., upregulates) gene expression. For this analysis, we focused on the In/B to pL G1 clusters, before the spermatogenic cells have undergone the major MEIOC-dependent transcriptomic shift observed in the scRNA-seq pseudotime analysis (Fig. 1A,B). Among the 48 MEIOC-YTHDC2-RBM46 targets downregulated by MEIOC within the In/B to pL G1 clusters, we identified eight mRNAs encoding transcriptional repressors: *Cbx1*, *E2f6*, *Hnrnpab*, *Mga*, *Pbrm1*, *Rad21*, *Sp3*, and *Suz12* (Fig. 3C,D). Strikingly, *Cbx1*, *E2f6* and *Mga* all encode subunits of the noncanonical Polycomb Repressive Complex (PRC) 1.6 (Fig. S7A, S9A), representing a statistically significant enrichment for the complex’s subunits (one-tailed hypergeometric test, *P* = 3.55E-05). *E2f6* and *Mga* are particularly attractive candidates because they are sequence-specific DNA-binding subunits of PRC1.6 required to repress meiosis-specific genes in somatic and embryonic stem cells (Pohlers et al. 2005; Dahlet et al. 2021; Kehoe et al. 2008; Maeda et al. 2013; Suzuki et al. 2016; Kitamura et al. 2021; Uranishi et al. 2021).

We hypothesized that MEIOC-YTHDC2-RBM46’s inhibition of *E2f6* and *Mga* was impacting gene expression during spermatogenesis. To test this, we reanalyzed ChIP-seq and RNA-seq datasets from mouse embryonic stem cells (Stielow et al. 2018; Qin et al. 2021; Dahlet et al. 2021) to identify genes that are directly repressed by E2F6 or MGA (see Materials and Methods; Fig. S8A,B). As E2F6 and MGA cooperate to repress expression of an overlapping set of genes (Dahlet et al. 2021) (Fig. S8C), we classified genes as repressed by both E2F6 and MGA, repressed by only E2F6, or repressed by only MGA. Genes repressed by both E2F6 and MGA, but not those repressed by either protein alone, were overrepresented among MEIOC-upregulated transcripts beginning in the mitotic In/B clusters, persisting through the mitosis-to-meiosis transition (Fig. 3E). This enrichment was not seen in MEIOC-downregulated transcripts (Fig. 3E). The bulk RNA-seq analysis of preleptotene-enriched testes largely confirmed these results, while also revealing that MEIOC-upregulated genes were enriched for genes repressed by only E2F6 and those repressed by only MGA (Fig. S9B), likely because this analysis included lowly expressed genes that were not detected in the scRNA-seq analysis. Therefore, MEIOC’s repression of E2F6 and MGA upregulates gene expression as early as in mitotic spermatogonia.

We also examined whether MEIOC’s repression of E2F6 and MGA leads to downregulated gene expression in spermatogenic cells. For this analysis, we used the mouse embryonic stem cell datasets to classify genes as activated by E2F6 and MGA, activated by only E2F6, or activated by only MGA. Genes activated by only MGA showed modest enrichment in MEIOC-downregulated genes within limited set of clusters (pL lS and L; Fig. S8D). Neither MEIOC-upregulated nor -downregulated transcripts were enriched for genes activated by both E2F6 and MGA or by only E2F6 (Fig. S8D). The bulk RNA-seq analysis of preleptotene-enriched testes confirmed these results (Fig. S9B). Thus, MEIOC’s repression of E2F6 and MGA does not lead to broad downregulation of gene expression. Instead, MEIOC’s repression of E2F6 and MGA primarily derepresses gene expression.

As many MEIOC-upregulated transcripts are meiotic cell cycle factors not bound by MEIOC-YTHDC2-RBM46 (Fig. 1D, 2F), we hypothesized that repression of E2F6 and MGA upregulates these meiotic genes. To test this, we asked whether E2F6- and MGA-repressed genes are enriched among meiotic cell cycle transcripts upregulated by MEIOC. We again focused this analysis on the In/B to pL G1 clusters, before germ cells have not undergone major MEIOC-dependent transcriptomic changes. We found that 13 of 17 MEIOC-upregulated meiotic cell cycle transcripts are targeted for repression by E2F6 and/or MGA (Fig. 3F). These observations were also confirmed in the bulk RNA-seq analysis of preleptotene-enriched testes (Fig. S9C). Therefore, MEIOC’s destabilization of *E2f6* and *Mga* mRNAs de-represses meiosis-associated gene expression beginning in mitotic spermatogonia.

### MEIOC indirectly activates transcriptional regulator STRA8-MEIOSIN to enhance spermatogenic cells’ competence to transition from mitosis to meiosis

**Figure 4:**
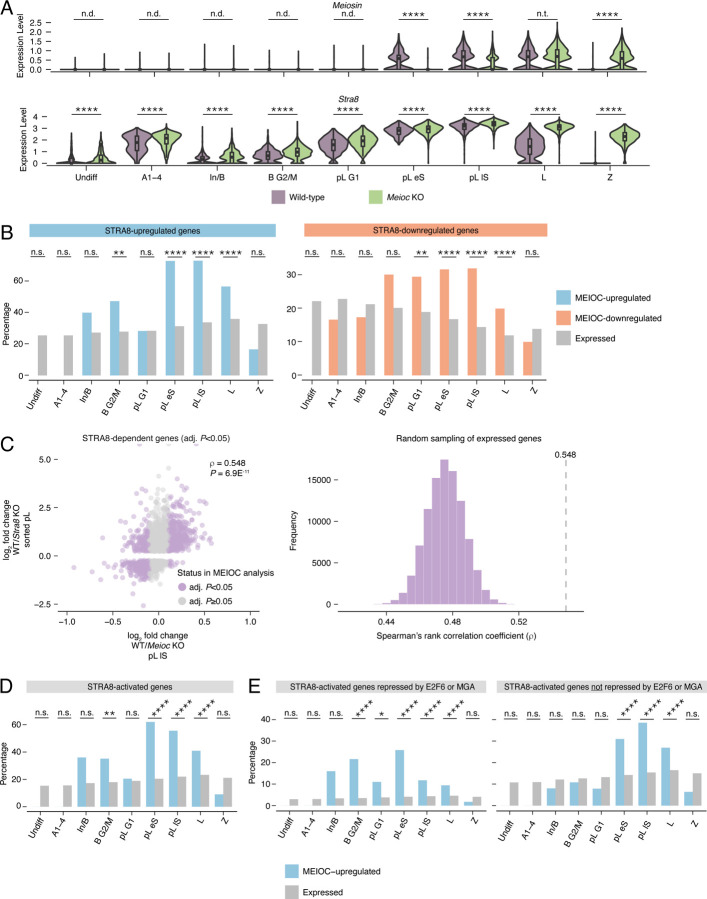
MEIOC’s derepression of *Meiosin* gene expression enhances activation of the STRA8-MEIOSIN transcriptional program. A: Expression levels of *Meiosin* and *Stra8* in wild-type and *Meioc* KO in all germ cell clusters. Clusters marked as “not done” (n.d.) did not meet expression thresholds set for statistical testing. B: Percentage of STRA8-upregulated and -downregulated genes in MEIOC-upregulated, -downregulated, and expressed genes from scRNA-seq analysis. STRA8-upregulated and -downregulated genes were identified via reanalysis of bulk RNA-seq data from wild-type and *Stra8* KO sorted preleptotene spermatocytes from Kojima et al., 2019. C: Left panel, correlation between MEIOC-dependent changes from scRNA-seq analysis of pL lS cluster and STRA8-dependent changes from bulk RNA-seq analysis of sorted preleptotene spermatocytes. Analysis was limited to genes that were statistically dependent on STRA8 (adjusted *P*<0.05). Right panel, distribution of correlations for gene sets obtained by random sampling of genes expressed in the scRNA-seq pL lS cluster and bulk RNA-seq sorted preleptotene spermatocytes. D: Percentage of STRA8-activated genes in MEIOC-upregulated and expressed genes from scRNA-seq analysis. STRA8-activated genes were identified as those genes with STRA8-bound promoters (as identified by Kojima et al. (2019) via STRA8-FLAG ChIP-seq in preleptotene-enriched testes) and upregulated by STRA8 (as identified by reanalysis of bulk RNA-seq data from wild-type and *Stra8* KO sorted preleptotene spermatocytes from Kojima et al., 2019). E: Percentage of STRA8-activated genes repressed by E2F6 or MGA (left), as well as STRA8-activated genes not repressed by E2F6 or MGA (right), in MEIOC-upregulated and expressed genes. *, adj. *P*<0.05; **, adj. *P*<0.01; ***, adj. *P*<0.001; ****, adj. *P*<0.0001; n.s., not significant; n.t., not tested (comparison was excluded from statistical testing because log_2_ fold change> −0.1 and <0.1); n.d., not detected (transcript expressed in <25% cells in each population being compared).

In mouse embryonic stem cells, E2F6 and MGA directly repress *Meiosin* (*Gm4969*) (Uranishi et al. 2021) (Fig. S8A,B). Consistent with MEIOC’s destabliization of *E2f6* and *Mga* mRNA, we found that MEIOC upregulates *Meiosin* expression at both the transcript and protein levels during the transition from mitosis to meiosis (Fig. 4A, S4C, S10A). By meiotic prophase I, *Meioc*-null germ cells exhibited delayed upregulation of *Meiosin* expression (Fig. 4A). We confirmed that *Meiosin* expression is also increased by YTHDC2 and RBM46, based on previously reported analyses of bulk RNA-seq data from postnatal testes (Jain et al. 2018; Peart et al. 2022). MEIOC does not bind *Meiosin* mRNA, nor does MEIOC affect other known regulators of *Meiosin* gene expression (i.e., retinoic acid-dependent transcription and DMRT1 activity; Fig. S10, S11; see Supplemental Information) (Ishiguro et al. 2020). Based on these data, MEIOC’s repression of *E2f6* and *Mga* derepresses *Meiosin* gene expression during the mitosis-to-meiosis transition.

STRA8-MEIOSIN functions as an obligate heterodimer that transcriptionally activates gene expression during the transition from mitosis to meiosis (Ishiguro et al. 2020; Kojima et al. 2019). Here, we hypothesized that MEIOC’s upregulation of *Meiosin* gene expression induces STRA8-MEIOSIN-mediated transcriptional changes. We tested this hypothesis in three ways.

First, we asked if, during the mitosis-to-meiosis transition, MEIOC-dependent genes are enriched for STRA8-dependent genes. We defined these STRA8-dependent genes by bulk RNA-seq data from wild-type and *Stra8*-null preleptotene spermatocytes isolated via synchronization and sorting (Kojima et al. 2019). We found that STRA8-upregulated genes were enriched among MEIOC-upregulated genes during the mitosis-to-meiosis transition (pL eS and lS clusters) and in meiotic prophase I (L cluster; Fig. 4B). Similarly, STRA8-downregulated transcripts were enriched among MEIOC-downregulated transcripts at these same stages (Fig. 4B). The bulk RNA-seq analysis of preleptotene-enriched testes revealed similar results (Fig. S12A).

Second, using STRA8-dependent genes, we correlated the transcript abundance changes from the MEIOC scRNA-seq and STRA8 bulk RNA-seq datasets. STRA8-dependent genes exhibited a statistically significant correlation between the MEIOC and STRA8 datasets that peaked during the mitosis-to-meiosis transition (pL eS and lS clusters; Fig. 4C, S13A, S14A). STRA8-dependent genes similarly exhibited a robust correlation between the MEIOC bulk RNA-seq dataset from preleptotene-enriched testes and the STRA8 dataset (Fig. S12B). Therefore, MEIOC supports STRA8-MEIOSIN-mediated changes in transcript abundance.

Third, we asked if MEIOC-dependent genes are enriched for genes directly activated by STRA8-MEIOSIN. We defined STRA8-activated genes using ChIP-seq results from testes enriched for preleptotene spermatocytes (Kojima et al. 2019) with the STRA8 bulk RNA-seq analysis (see Materials and Methods). We found that MEIOC-upregulated genes were enriched for STRA8-activated genes during the mitosis-to-meiosis transition (pL eS and lS clusters; Fig. 4D). This enrichment was also evident in the bulk RNA-seq analysis of preleptotene-enriched testes (Fig. S12C). Furthermore, the top motif identified by de novo motif analysis of MEIOC-upregulated genes from the bulk RNA-seq analysis matched the STRA8-MEIOSIN binding motif (Fig. S12D). In total, ~60–70% of MEIOC-upregulated genes are directly activated by STRA8-MEIOSIN.

Together, these analyses demonstrate that MEIOC indirectly activates the STRA8-MEIOSIN transcriptional regulator during the transition from mitosis to meiosis, facilitating a major transcriptomic shift at the meiotic G1/S transition (Fig. 1A). With this new molecular insight, we revisited our interpretation of the *Meioc*-null phenotype. Based on prior histological analysis, *Meioc*-null spermatogenic cells accumulate at the preleptotene stage and are delayed in entering the leptotene stage (Soh et al. 2017; Abby et al. 2016). This is phenotypically distinct from spermatogenic cells that lack the developmental competence to transition from mitosis to meiosis, as *Stra8*-null or *Meiosin*-null cells on the same genetic background arrest at the preleptotene stage (Ishiguro et al. 2020; Kojima et al. 2019; Anderson et al. 2008b). Given that *Meioc*-null germ cells still exhibit some *Meiosin* expression, we interpret the *Meioc*-null phenotype as delayed acquisition of the developmental competence to transition from mitosis to meiosis. Therefore, MEIOC enhances spermatogenic cells’ competence to initiate meiosis by activating the STRA8-MEIOSIN transcriptional regulator.

### MEIOC derepresses meiotic gene expression before activating STRA8-MEIOSIN

We observed that many STRA8-activated genes are also repressed by E2F6 and/or MGA (Fig. S12E). As MEIOC represses E2F6 and MGA in mitotic spermatogonia and activates STRA8-MEIOSIN during the mitosis-to-meiosis transition, we hypothesized that MEIOC-regulated genes exhibit temporally distinct regulation, dependent on whether they were targeted by only STRA8-MEIOSIN or by both E2F6-MGA and STRA8-MEIOSIN. Specifically, we predicted that genes experimentally defined as E2F6 and/or MGA-repressed and STRA8-activated would be upregulated by MEIOC starting in mitotic spermatogonia, coincident with MEIOC’s destabilization of *E2f6* and *Mga*. By contrast, STRA8-activated genes that were not repressed by E2F6 or MGA would only be upregulated by MEIOC later during the mitosis-to-meiosis transition, coincident with MEIOC’s upregulation of *Meiosin* gene expression. Indeed, consistent with our predictions, STRA8-activated, E2F6-MGA-repressed genes were enriched among MEIOC-upregulated genes in the mitotic B G2/M cluster as well as during the mitosis-to-meiosis transition (pL G1, eS, and lS clusters; Fig. 4E). By contrast, STRA8-activated genes that were not repressed by E2F6 or MGA exhibited enrichment during the pL eS and lS clusters (Fig. 4E). Both gene sets were also enriched among MEIOC-upregulated genes in the bulk RNA-seq analysis (Fig. S12F). Therefore, during late transit amplification, MEIOC derepresses a set of meiotic genes targeted by E2F6 and MGA; then during the mitosis-to-meiosis transition, MEIOC increases the expression of these and other meiotic genes by activating STRA8-MEIOSIN.

**Figure 5:**
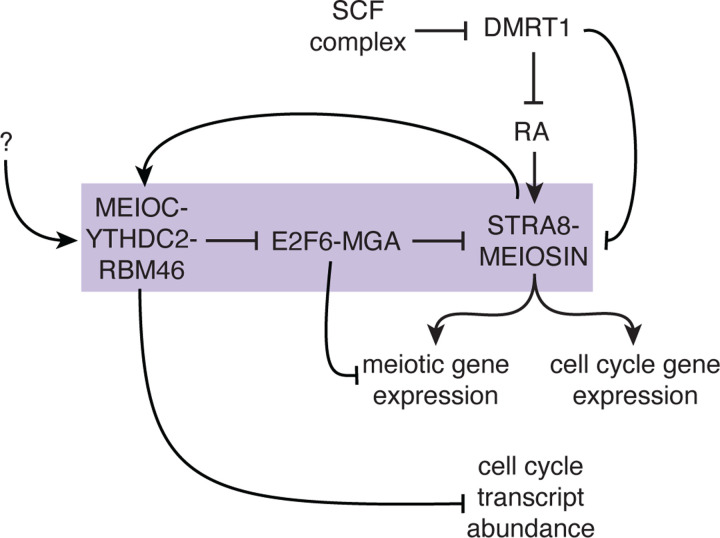
Model for MEIOC-YTHDC2-RBM46 enhancing spermatogenic cells’ competence to transition from mitosis to meiosis in response to retinoic acid. MEIOC-YTHDC2-RBM46 destabilize *E2f6* and *Mga* mRNA and thereby inhibit E2F6 and MGA’s repression of transcription at genes involved in meiosis, including *Meiosin*. In parallel, the SCF complex degrades DMRT1 and consequently inhibits DMRT1’s repression of retinoic acid (RA)-dependent transcription as well as *Stra8* and *Meiosin* gene expression. Retinoic acid activates *Stra8* and *Meiosin* gene expression. This activates the STRA8-MEIOSIN transcription factor, which drives the transcription of cell cycle genes as well as meiotic genes, many of which were previously repressed by E2F6 and MGA. MEIOC’s repression of cell cycle transcripts also contributes to the establishment of a meiosis-specific cell cycle program in meiotic prophase I. The question mark denotes undefined molecular regulation that activates MEIOC-YTHDC2-RBM46 before retinoic acid activates STRA8-MEIOSIN transcriptional activity and the transition from mitosis to meiosis. Box highlights the novel regulation identified in this study that facilitates competence for the mitosis-to-meiosis transition.

In total, MEIOC-YTHDC2-RBM46’s destabilization of *E2f6* and *Mga* mRNAs in late mitotic spermatogenic cells derepresses E2F6-MGA-targeted genes, including *Meiosin* and other meiosis-associated genes. In turn, this activity supports activation of the STRA8-MEIOSIN transcriptional regulator by retinoic acid during the transition from mitosis to meiosis (Fig. 5). As STRA8-MEIOSIN is the molecular vehicle by which retinoic acid induces the meiotic G1/S transition, MEIOC’s regulation of the STRA8-MEIOSIN complex enhances spermatogenic cells’ competence to undergo the mitosis-to-meiosis transition.

## DISCUSSION

Here we use scRNA-seq, as well as bulk RNA-seq of developmentally synchronized and histologically staged spermatogenesis, to demonstrate that *Meioc*-null spermatogenic cells developmentally diverge from their wild-type counterparts during the meiotic G1/S transition, earlier than previously appreciated. We find that MEIOC is required to derepress expression of meiotic genes, including the meiotic transcriptional regulator *Meiosin*. In turn, MEIOC increases the expression of genes targeted by STRA8-MEIOSIN, which drives the mitosis-to-meiosis transition. Therefore, MEIOC enhances differentiating spermatogonia’s competence to activate the meiotic transcriptional regulator and transition from mitosis to meiosis in response to retinoic acid.

We find that MEIOC-YTHDC2-RBM46 destabilizes its mRNA targets, as has been suggested previously based on analyses of transcript abundance (Saito et al. 2022; Hsu et al. 2017; Soh et al. 2017; Qian et al. 2022). In the current study, we distinguished changes in transcription vs. transcript stability via two approaches. First, scRNA-seq allowed us to identify and analyze spermatogenic cells that had been impacted by MEIOC before the onset of major transcriptional changes. Second, we applied a specialized pipeline to distinguish between transcriptional rate and RNA stability in bulk RNA-seq data of preleptotene-enriched testes (Alkallas et al. 2017). Both complementary methods confirm that MEIOC-YTHDC2-RBM46 reduces the stability of its target transcripts. Transcript stability may be the primary mechanism by which MEIOC-YTHDC2-RBM46 regulates its targets, as a recent study found that YTHDC2 does not affect translation in postnatal testes (Saito et al. 2022). Whether MEIOC-YTHDC2-RBM46 impacts transcript localization remains to be examined.

Beginning in mitotic spermatogonia, MEIOC-YTHDC2-RBM46 increases the abundance of meiosis-associated transcripts without directly binding many of these transcripts. Here, we demonstrate that these transcript abundance changes are driven by changes in transcription rather than mRNA stability (Fig. 3B). Further, we provide a two-tiered mechanism for these indirect changes in transcript abundance. First, MEIOC-YTHDC2-RBM46 binds to and destabilizes *E2f6* and *Mga* mRNAs, which encode transcriptional repressors whose genomic targets are enriched for meiosis-associated genes (Pohlers et al. 2005; Dahlet et al. 2021; Kehoe et al. 2008; Maeda et al. 2013; Suzuki et al. 2016; Kitamura et al. 2021; Uranishi et al. 2021). Second, MEIOC-YTHDC2-RBM46’s repression of *E2f6* and *Mga* derepresses *Meiosin* gene expression and activates STRA8-MEIOSIN. This transcriptional regulator then activates meiotic gene expression and the meiotic G1/S transition. Thus, MEIOC-YTHDC2-RBM46’s repression of a transcriptional repressor confers developmental competence to transition from mitosis to meiosis.

In embryonic oocytes, loss of PRC1 activity via genetic ablation of *Ring1* and *Rnf2* causes premature meiosis due to precocious expression of *Stra8* and other meiosis-associated genes (Yokobayashi et al. 2013). Therefore, it is plausible that, during spermatogenesis, PRC1 also regulates gene expression and the timing of meiotic initiation, but this has not been directly tested. In addition, the germline activity of PRC1.6 subunits E2F6 and MGA remains poorly characterized. While *E2f6*-null mice are fertile, spermatogenesis was reported as disrupted without detailed characterization (Storre et al. 2002). Genetic ablation of *Mga* causes embryonic lethality, precluding any analysis of spermatogenesis (Burn et al. 2018; Washkowitz et al. 2015). Therefore, additional genetic studies are needed to identify E2F6 and MGA’s function during spermatogenesis and to determine the extent to which loss of *E2f6* and *Mga* can rescue the developmental competence of *Meioc*-null spermatogenic cells. In addition, it is possible that other transcriptional repressors targeted by MEIOC-YTHDC2-RBM46 may also control *Meiosin* gene expression.

As some *Meioc*-null spermatogenic cells upregulate *Meiosin* gene expression and enter meiotic prophase I along a delayed timeline, MEIOC is not strictly required for the competence to transition from mitosis to meiosis. It is possible that residual activity from YTHDC2-RBM46 in the absence of MEIOC can support meiotic entry in some cells; alternatively, the DMRT1-mediated acquisition of competence may support mitotic spermatogonia’s competence to transition from mitosis to meiosis along this delayed timeline. Regardless, our model establishes that the *Meioc*, *Ythdc2*, and *Rbm46*-null phenotype of delayed entry into meiotic prophase I is the result of reduced STRA8-MEIOSIN transcriptional activation and is therefore the less severe manifestation of the arrest at the mitotic-to-meiosis transition, before meiotic prophase I, exhibited by *Stra8* and *Meiosin*-null spermatogenic cells on an inbred C57BL/6 background. Intriguingly, *Stra8*-null spermatogenic cells on a mixed genetic background exhibit the less severe phenotype similar to *Meioc*, *Ythdc2*, and *Rbm46*-null spermatogenic cells. Our analyses of the *Meioc*-null phenotype suggest that on a mixed genetic background, the *Stra8*-null spermatogenic cells contain residual transcriptional activation of some meiotic gene expression, potentially due to MEIOSIN acting as a homodimer, but further work is needed to test this prediction.

Our model for mammalian meiotic initiation exhibits parallels, without direct orthology or homology, to the molecular network that governs budding yeast meiotic initiation. Specifically, repression of a transcriptional repressor (Rme1p) via mRNA destabilization activates expression of the key transcription factor (Ime1p) that governs meiotic entry. Therefore, in both unicellular eukaryotes and multicellular organisms, destabilization of a transcriptional repressor at the transcript level controls activation of the meiotic transcriptional program.

We have placed transcriptional activation by STRA8-MEIOSIN and post-transcriptional repression of mRNA by MEIOC-YTHDC2-RBM46 in a positive feedback loop that facilitates the mitosis-to-meiosis transition (Fig. 5). This model also generates a new question: how is MEIOC-YTHDC2-RBM46 activated before retinoic acid activates STRA8-MEIOSIN and the transition from mitosis to meiosis (Fig. 5)? One possibility is illustrated by MEIOC and YTHDC2’s *Drosophila* homologs Bam and Bgcn, which (translationally) repress their mRNA targets in late mitotic spermatogonia to facilitate the transition from mitosis to meiosis (Insco et al. 2009, 2012). This transition requires the accumulation of Bam protein to a critical threshold (Insco et al. 2009). Whether mammalian MEIOC must similarly accumulate to a critical threshold to activate the MEIOC-YTHDC2-RBM46 complex requires further investigation.

In conclusion, by functioning as a destabilizer of its mRNA targets, MEIOC-YTHDC2-RBM46 represses transcriptional repressors E2F6 and MGA and thereby allows spermatogenic cells to activate *Meiosin* expression in response to retinoic acid. Consequently, this activates the key meiotic transcriptional regulator STRA8-MEIOSIN, which amplifies expression of meiosis- and cell cycle-associated genes and drives the meiotic G1/S transition. In total, the post-transcriptional activity of MEIOC-YTHDC2-RBM46 enhances the activity of the meiotic transcriptional regulator. This regulation, acting as a second, parallel mechanism to the SCF complex’s degradation of DMRT1, enhances mitotic spermatogonia’s competence to transition from mitosis to meiosis in response to retinoic acid.

## MATERIALS AND METHODS

### Animals

All experiments involving mice were performed in accordance with the guidelines of the Massachusetts Institute of Technology (MIT) Division of Comparative Medicine and Cincinnati Children’s Hospital Medical Center (CCHMC) Division of Veterinary Services, which are overseen by their respective Institutional Animal Care and Use Committees (IACUC). The animal care programs at MIT/Whitehead Institute and CCHMC are accredited by the Association for Assessment and Accreditation of Laboratory Animal Care, International (AAALAC) and meet or exceed the standards of AAALAC as detailed in the Guide for the Care and Use of Laboratory Animals. This research was approved by the MIT IACUC (no. 0617-059-20) and CCHMC IACUC (no. 2022-0061).

Mice carrying the *Meioc*-null allele *Meioc*^*tm1.1Dpc*^ (Soh et al. 2017) were backcrossed to C57BL/6N (B6N) from Taconic Biosciences for at least 10 generations. Mice used for scRNA-seq experiments were also heterozygous for *Hspa2*^*tm1Dix*^ (RRID: IMSR_JAX:013584;(Dix et al. 1996)) and homozygous for *Gt(ROSA)26Sor*^*tm9(CAG-tdTomato)Hze*^ (*ROSA26*^*tdTomato*^; RRID:IMSR_JAX:007909; (Madisen et al. 2010)) with the floxed stop codon intact; both of these genotypes exhibit normal spermatogenesis.

### 10x Genomics single-cell RNA-seq

Single-cell sequencing libraries were prepared and sequenced in two batches, with each batch containing one wild-type and one *Meioc*-null pup at P15. One P15 testis per pup was enzymatically dissociated into single cells (see Supplemental Information) and resuspended in 0.05% bovine serum albumin (BSA) in phosphate buffered saline (PBS) for a target concentration of 1000 cells per microliter. Cell suspensions were loaded onto the Chromium Controller, aiming for recovery of 10,000 cells per sample. Libraries were generated using the Chromium Next GEM Single Cell 3ʹ v3.1 (10x Genomics), according to manufacturer’s instructions, and sequenced as 150 bp paired-end reads on an Illumina NovaSeq 6000 system with an S4 flow cell.

### Analysis of 10x Genomics scRNA-seq data

Alignment, filtering, barcode counting, and UMI counting were done via *count* function in Cell Ranger v.4.0.0 with default settings using Cell Ranger’s mm10-2020-A reference package (i.e., the GRCm38/mm10 mouse genome assembly with GENCODE vM23/Ensembl 98 annotation). Using Seurat v.3.2.3 (Stuart et al. 2019), cells were filtered for less than 10% mitochondrial reads, more than 1000 detected features, and a doublet score (generated by the *bcds* in scds v.1.2.0) of less than 0.4. Using protein-coding genes, UMI counts from both wild-type and *Meioc*-null samples were integrated and clusters identified using the first 30 dimensions. The wild-type samples were used to assign cell types to clusters, first using cell type-enriched gene expression (Fig. S1B) and refined based on cell cycle phase using the *CellCycleScoring* function in Seurat (Fig. S1C,D). Clusters were merged as needed, and clusters with less than five cells were removed. Cell cycle designations of the preleptotene clusters were confirmed via gene expression patterns in wild-type cells independent of the gene:cell cycle phase pairings used by Seurat’s *CellCycleScoring* function (see Supplemental Information). These final cell type designations were then applied to the *Meioc*-null samples. An additional spermatocyte cluster (labeled Mut) was identified in the *Meioc*-null samples that was not identified in the wild-type samples. In addition to the germ cell and somatic clusters shown in Fig. S1A,B, one additional somatic cluster and one additional germ cell cluster were also identified but could not be assigned a cell type using marker expression. These unassigned somatic and germ cell clusters, along with all other somatic clusters, were excluded from subsequent analyses. See Supplemental Information for additional details on cluster-specific enrichment or depletion in wild-type cells and Gene Set Enrichment Analysis (GSEA).

The pseudotime trajectory was built in Monocle 3 v1.0.0 (Trapnell et al. 2014; Qiu et al. 2017a, 2017b) using the following procedure: the Seurat object containing germ cell clusters only was imported as a Monocle object; data were normalized and pre-processed (function *preprocess_cds()*; options: num_dim = 100); batch effects were removed (function *align_cds()*; options: alignment_group = "batch"), dimensionality reduction was done via UMAP (function *reduce_dimension()*); cells were clustered (function *cluster_cells()*); a principal graph was learned from the reduced dimension space (function *learn_graph()*); and cells were ordered by selecting the Undiff cluster as the starting branch of the trajectory (function *order_cells()*). Cluster cell-type assignments made in Seurat were maintained in the pseudotime trajectory.

For differential expression analysis between wild-type and *Meioc*-null germ cells log_2_ fold change was defined as wild-type over *Meioc*-null germ cells, such that the value reflects MEIOC’s activity in the unperturbed wild-type state. Differential expression analysis of scRNA-seq data was done on each germ cell cluster on genes with a minimum absolute log_2_ fold change of 0.1 and that were detected in at least 25% of either population using Seurat’s *FindMarkers* function (options: logfc.threshold = 0.1, min.pct = 0.25); *P* values were adjusted for multiple hypothesis testing of tested genes across all 9 germ cell clusters via the Bonferroni correction using the *p.adjust* function in R. Additional expressed genes (i.e., detected in at least 25% of cells in wild-type or *Meioc*-null cells per cluster) that failed to meet the log_2_ fold change threshold for differential expression testing were identified via the *FindMarkers* function (options: logfc.threshold=0, min.pct = 0.25), and their *P* values were marked as “nd” for differential expression testing “not done.”

Dot plots were generated using Seurat’s *DotPlot* function with parameter scale=FALSE to maintain average expression from wild-type and *Meioc*-null samples on the same scale.

### Synchronization of spermatogenesis

Spermatogenesis was synchronized using a protocol originally developed by Hogarth et al. (Hogarth et al. 2013) and modified by Romer et al. (Romer et al. 2018).

### Bulk RNA-seq analysis of preleptotene-enriched testes

Total RNA was extracted from 4 wild-type samples and 3 *Meioc*-null samples, using 1.5 synchronized testes from a single pup per sample. TRIzol Reagent (Thermo Fisher Scientific) was added to freshly thawed whole testes, and ERCC RNA ExFold RNA Spike-In Mix 1 and 2 (Thermo Fisher Scientific) were added to wild-type and *Meioc*-null samples, respectively, at a concentration of 1 μl of a 1:100 dilution of spike-in mix per 1 mg of testis tissue. (Spike-in mixes were ultimately not used for data analysis.) Total RNA was then isolated with chloroform following the manufacturer’s protocol, precipitated via isopropanol, and resuspended in RNase-free water. RNA-seq libraries were prepared with the TruSeq Stranded Total RNA kit with the Ribo-Zero Gold rRNA Removal kit. The barcoded libraries were pooled and sequenced with 50bp single-end reads on an Illumina HiSeq 2500 machine.

Reads were quality trimmed using cutadapt v1.8 (options: -q 30 --minimum-length 20 -b AGATCGGAAGAGC). Expression levels of all transcripts in the mouse Gencode Basic vM15 gene annotation were estimated using kallisto v0.44.0 (Bray et al. 2016) with sequence-bias correction (--bias) and strand-specific pseudoalignment (--rf-stranded). Quantified transcripts were filtered for protein-coding genes, transcript-level estimated counts and transcripts per million (TPM) values were summed to the gene level, and TPMs were renormalized to transcript-per-million units.

To identify the MEIOC-dependent differential expression program, read counts from kallisto were rounded to the nearest integer and then supplied to DESeq2 v1.26.0 (Love et al. 2014). Genes were filtered for a minimum TPM of 1 in at least 3 of 7 2S preleptotene samples, and differential expression/abundance was defined using a cutoff of adjusted *P* value < 0.05.

For analysis of transcript stability and transcriptional rates, reads were mapped to the mouse genome (mm10) with the GENCODE Basic vM15 gene annotation via STAR v2.7.1a (Dobin et al. 2013) (options: --outFilterMultimapNmax 1 --alignEndsType Extend5pOfRead1 -- outFilterMismatchNmax 2 --outSAMattributes None). All other parameters were set to default. Counts were quantified by htseq v0.11.0 (options: -m union --stranded=reverse) at the gene level (-type gene) and exon level (-type exon), and intron levels were calculated as gene-level counts minus exon-level counts. Changes in transcript stability, transcriptional rate, and abundance were calculated for each gene for each sample using REMBRANDTS (Alkallas et al. 2017) with a stringency of 0.80 and linear bias mode. The change between wild-type and *Meioc*-null samples was then calculated in R.

### Re-analysis of STRA8 RNA-seq dataset

RNA-seq data from *Stra8*-null and *Stra8*-heterozygote (i.e., phenotypically wild-type) preleptotene spermatocytes (NCBI GEO GSE115928; Kojima et al. 2019) were reanalyzed. Genes with STRA8-bound promoters identified by ChIP-seq in testes enriched for preleptotene spermatocytes were extracted from Supplementary File 2 (Kojima et al. 2019) (https://doi.org/10.7554/eLife.43738.028). See Supplemental Information for details.

### Gene Ontology analysis of scRNA-seq and bulk RNA-seq data

Within each scRNA-seq cluster, MEIOC-upregulated (log_2_ fold change > 0.1 and adjusted *P* value < 0.05) and MEIOC-downregulated genes (log_2_ fold change < −0.1 and adjusted *P* value < 0.05) were separately tested for enrichment of Gene Ontology Biological Processes gene lists relative to a background of all expressed genes (i.e., genes expressed in at least 25% of wild-type or *Meioc*-null cells within that cluster). For bulk RNA-seq data, MEIOC-upregulated (log_2_ fold change > 0 and adjusted *P* value < 0.05) and MEIOC-downregulated genes (log_2_ fold change < − 0.1 and adjusted *P* value < 0.05) were similarly tested against a background of all expressed genes (i.e., a minimum TPM of 1 in at least 3 of 7 2S preleptotene samples). These analyses were done via R package clusterProfiler v3.0.4 using the *enrichGO* function (options: OrgDb = "org.Mm.eg.db", ont = "BP", pvalueCutoff = 0.05, readable = T, pAdjustMethod = "BH"). For the scRNA-seq data, the average log_2_ fold change for select upregulated and downregulated genes that fell under GO terms “meiotic cell cycle” (GO:0051321) and “mitotic cell cycle” (GO:0000278), respectively, were graphed via heatmap to show gene expression changes between wild-type and *Meioc*-null cells across scRNA-seq germ cell clusters.

### Re-analysis of MEIOC RIP-seq dataset and comparison to YTHDC2 and RBM46 CLIP datasets

MEIOC RIP-seq from P15 testes (NCBI GEO GSE96920; Soh et al. 2017) were reanalyzed, with additional details in Supplemental information.

YTHDC2-bound mRNAs identified via CLIP-seq in P8 and P10 testes were extracted from Tables S1 and S2 (Saito et al. 2022) (http://genesdev.cshlp.org/content/suppl/2022/01/19/gad.349190.121.DC1/Supplemental_Tables.xlsx).

RBM46 eCLIP data from P12-P14 testes (NCBI GEO GSE197282) (Qian et al. 2022) were reanalyzed, with additional details in Supplemental Information.

### Re-analysis of E2F6 and MGA ChIP-seq datasets and RNA-seq datasets from ESCs

The following datasets were reanalyzed here: E2F6 ChIP-seq and input data from wild-type and *E2f6*-knockout mouse ESCs (NCBI GEO GSE149025) (Dahlet et al. 2021); MGA and IgG ChIP-seq from wild-type mouse ESCs (ArrayExpress E-MTAB-6007) (Stielow et al. 2018); mouse ESC RNA-seq data from wild-type and *E2f6*-knockout samples (NCBI GEO GSE149025) (Dahlet et al. 2021); and wild-type and *Mga*-knockout samples (NCBI GEO GSE144141) (Qin et al. 2021). See Supplemental Information for details.

## Supplementary Material

Supplement 1

Supplement 2
