# Supplementary material for: Post-transcriptional repression of mRNA enhances competence to transit from mitosis to meiosis in mouse spermatogenic cells
Source: bioRxiv. 2023 Sep 22:2023.09.20.557439. Preprint. [Version 1] doi: 10.1101/2023.09.20.557439 (PMC10541148; doi:10.1101/2023.09.20.557439)
Supplement: Supplement 1 [file media-1.pdf]

# Figure S1

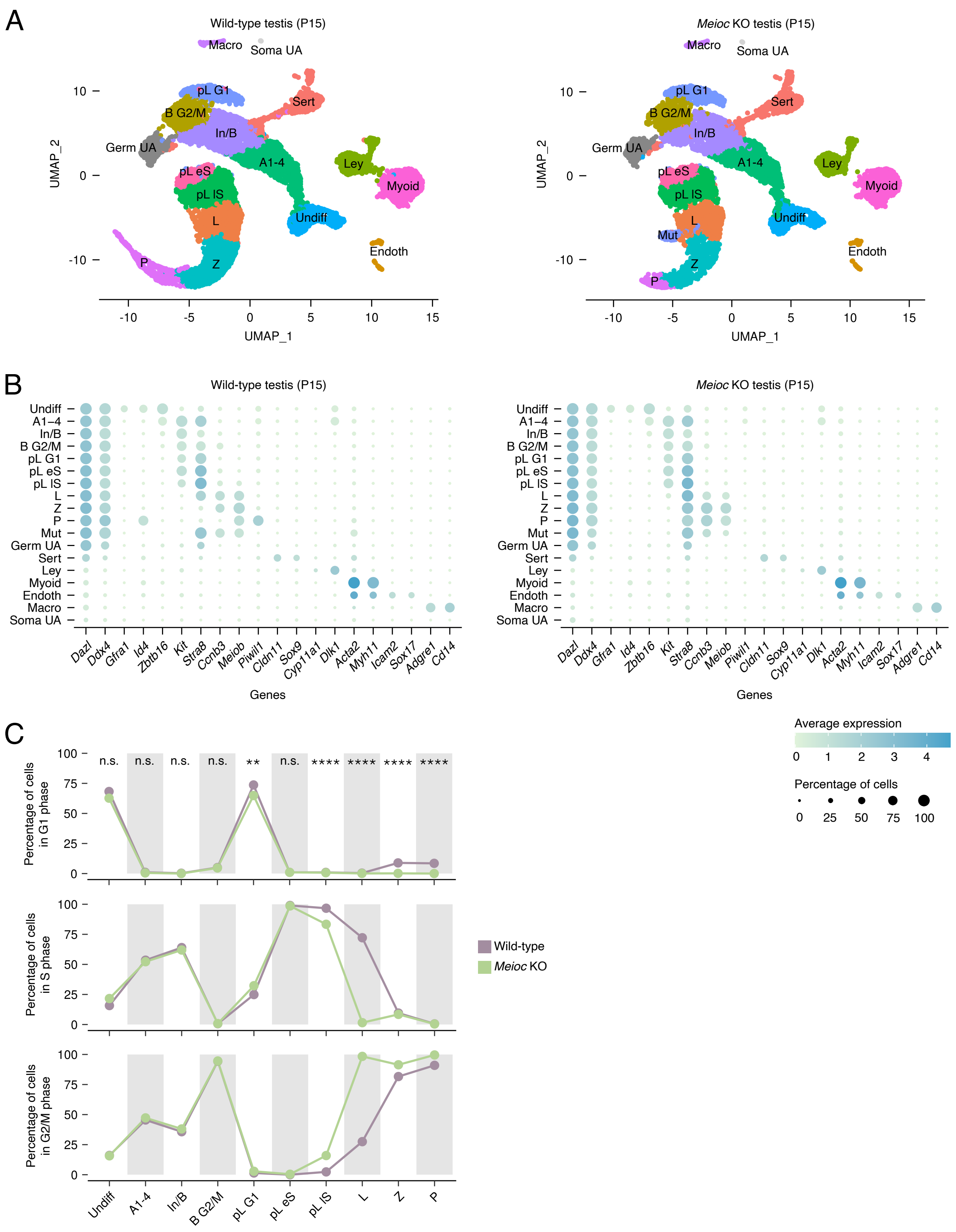

A
Wild-type testis (P15)
Meioc KO testis (P15)
Macro
Soma UA
pL G1
Sert
B G2/M
In/B
Germ UA
A1-4
Ley
pL eS
pL IS
Myoid
Undiff
L
Mut
Endoth
Z
P
UMAP_1
UMAP_2
B
Genes
Dazl
Ddx4
Gfra1
Id4
Zbtb16
Kit
Stra8
Ccnb3
Meiob
Piwil1
Cldn11
Sox9
Cyp11a1
Dlk1
Acta2
Myh11
Icam2
Sox17
Adgre1
Cd14
Average expression
Percentage of cells
C
n.s.
**
****
Percentage of cells in G1 phase
Percentage of cells in S phase
Percentage of cells in G2/M phase
Wild-type
Meioc KO

Figure S1: Identification of germ cell subpopulations across the mitosis-to-meiosis transition via scRNA-seq.

A: UMAP of all clusters identified via Seurat from wild-type and *Meioc* KO P15 testes.

B: Dotplot of expression levels and percentage of cells for markers used to assign clusters.

C: Distribution of cell cycle phase per germ cell cluster from wild-type and *Meioc* KO samples.

*, adj. $P$<0.05; **, adj. $P$<0.01; ***, adj. $P$<0.001; adj. $P$<0.0001; n.s., not significant.

Figure S2

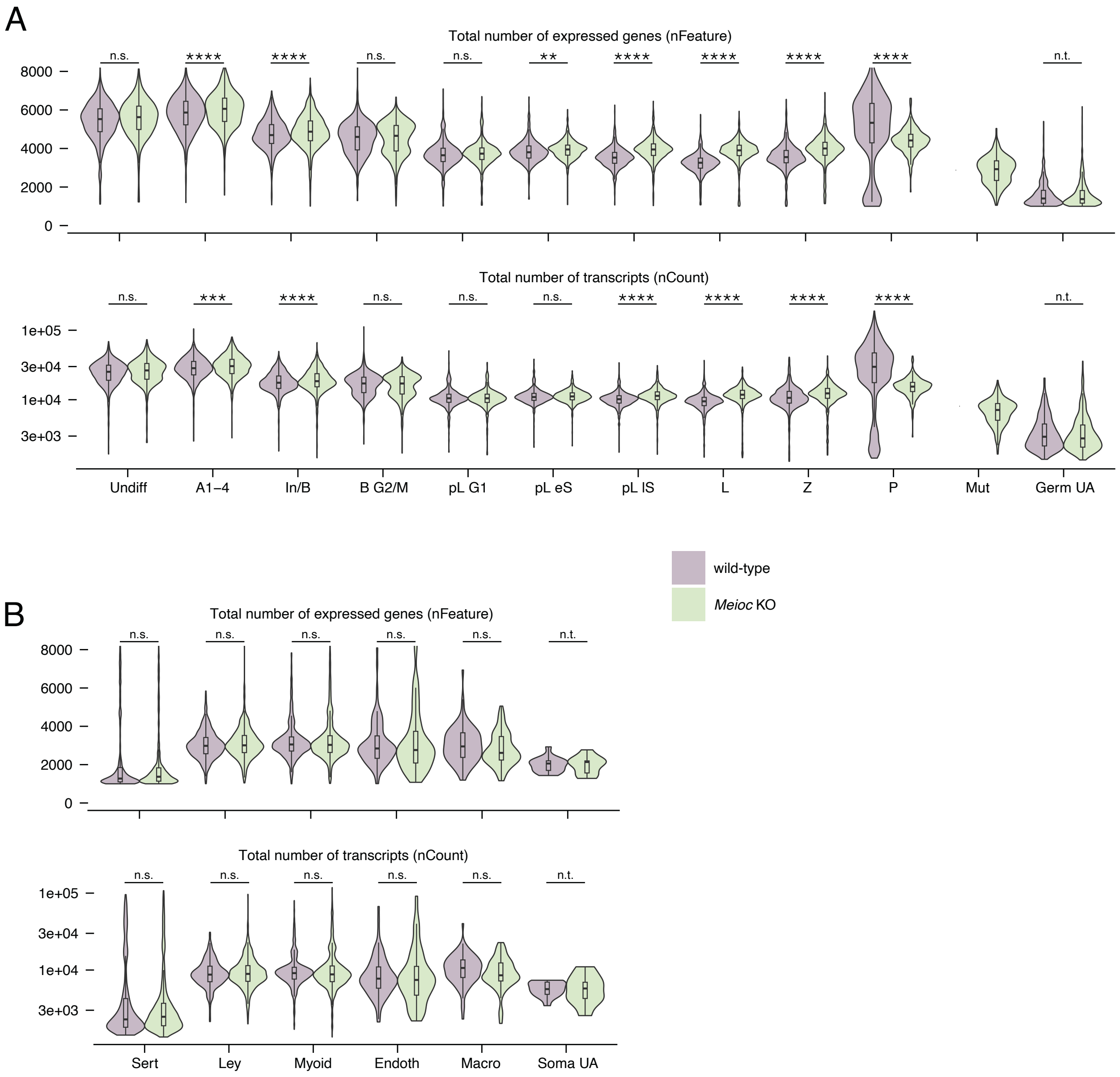


Figure S2: Features and counts in scRNA-seq data.

A: Total number of genes expressed (nFeature) and transcripts detected (nCount) in wild-type and *Meioc*-null cells for germ cell clusters.
B: Total number of genes expressed (nFeature) and transcripts detected (nCount) in wild-type and *Meioc*-null cells for somatic clusters.

*, adj. *P*<0.05; **, adj. *P*<0.01; ***, adj. *P*<0.001; ****, adj. *P*<0.0001; n.s., not significant; n.t., not tested, as populations may represent sequencing artifacts.

# Figure S3

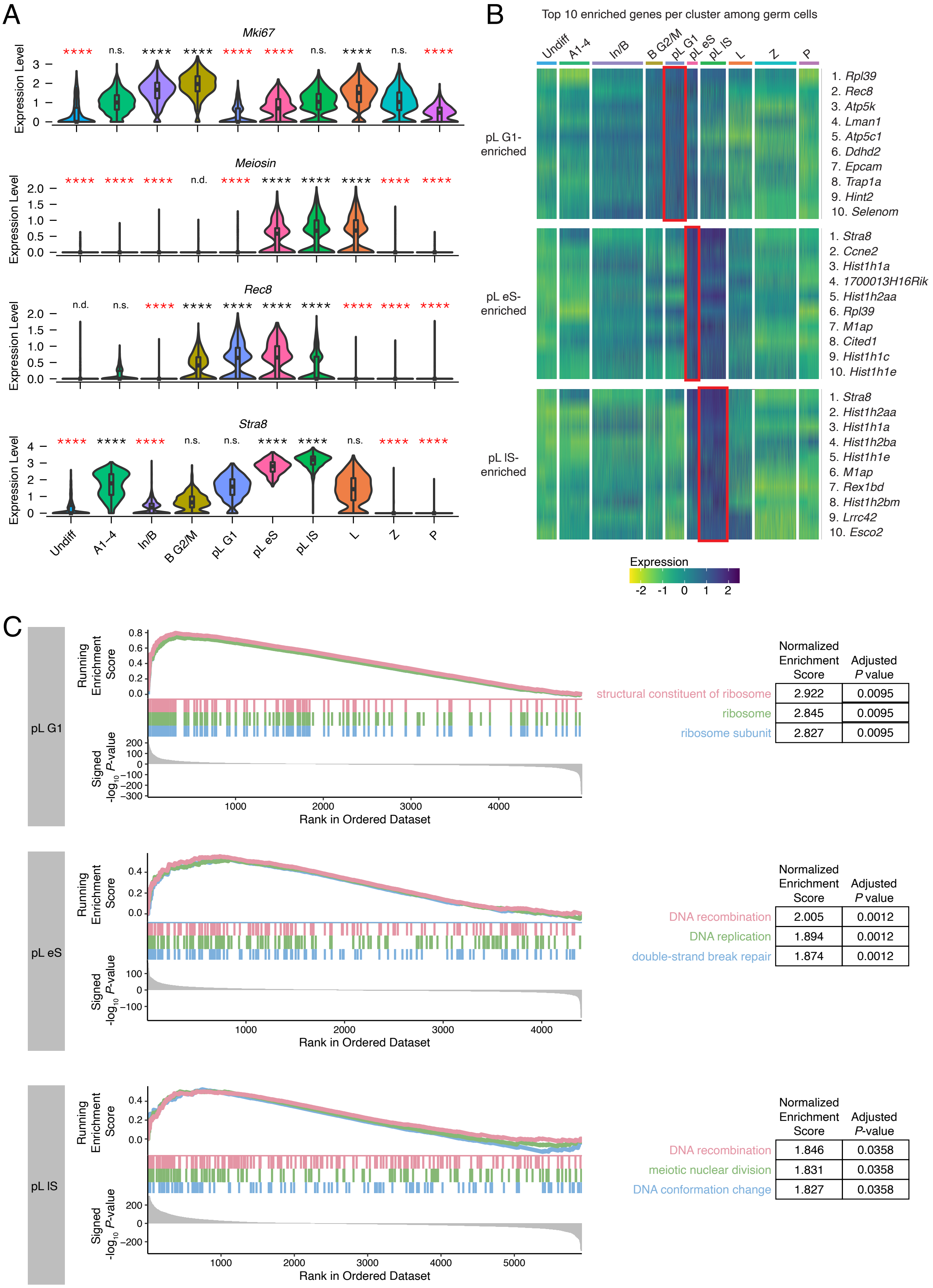
A
Mki67
Meiosin
Rec8
Stra8
Expression Level
****
n.s.
n.d.
Undiff
A1–4
In/B
B G2/M
pL G1
pL eS
pL lS
L
Z
P
B
Top 10 enriched genes per cluster among germ cells
pL G1-enriched
1. Rpl39
2. Rec8
3. Atp5k
4. Lman1
5. Atp5c1
6. Ddhd2
7. Epcam
8. Trap1a
9. Hint2
10. Selenom
pL eS-enriched
1. Stra8
2. Ccne2
3. Hist1h1a
4. 1700013H16Rik
5. Hist1h2aa
6. Rpl39
7. M1ap
8. Cited1
9. Hist1h1c
10. Hist1h1e
pL lS-enriched
1. Stra8
2. Hist1h2aa
3. Hist1h1a
4. Hist1h2ba
5. Hist1h1e
6. M1ap
7. Rex1bd
8. Hist1h2bm
9. Lrrc42
10. Esco2
Expression
-2 -1 0 1 2
C
pL G1
pL eS
pL lS
Running Enrichment Score
Signed -log10 P-value
Rank in Ordered Dataset
Normalized Enrichment Score
Adjusted P value
structural constituent of ribosome 2.922 0.0095
ribosome 2.845 0.0095
ribosome subunit 2.827 0.0095
DNA recombination 2.005 0.0012
DNA replication 1.894 0.0012
double-strand break repair 1.874 0.0012
Adjusted P-value
DNA recombination 1.846 0.0358
meiotic nuclear division 1.831 0.0358
DNA conformation change 1.827 0.0358

Figure S3: Distinguishing features among wild-type preleptotene clusters.

A: Wild-type expression levels of *Mki67*, *Meiosin*, *Rec8*, and *Stra8* for all germ cell clusters. Black and red asterisks designate enrichment or depletion, respectively, relative to all other germ cells. Clusters marked as “not done” (n.d.) did not meet expression thresholds set for statistical testing.

B: Heatmap of the top 10 enriched genes in the G1, early S, and late S preleptotene clusters.

C: Gene Set Enrichment Analysis (GSEA) for Gene Ontology (GO) terms for genes within each celltype cluster. Genes were ranked by the signed -log10(*P* value) for their level of enrichment/depletion within each cluster relative to all other germ cell clusters. Top three enriched GO terms for each cluster shown.

*, adj. $P<0.05$; **, adj. $P<0.01$; ***, adj. $P<0.001$; ****, adj. $P<0.0001$; n.s., not significant; n.d., not detected (transcript expressed in <25% cells in each population being compared).

# Figure S4

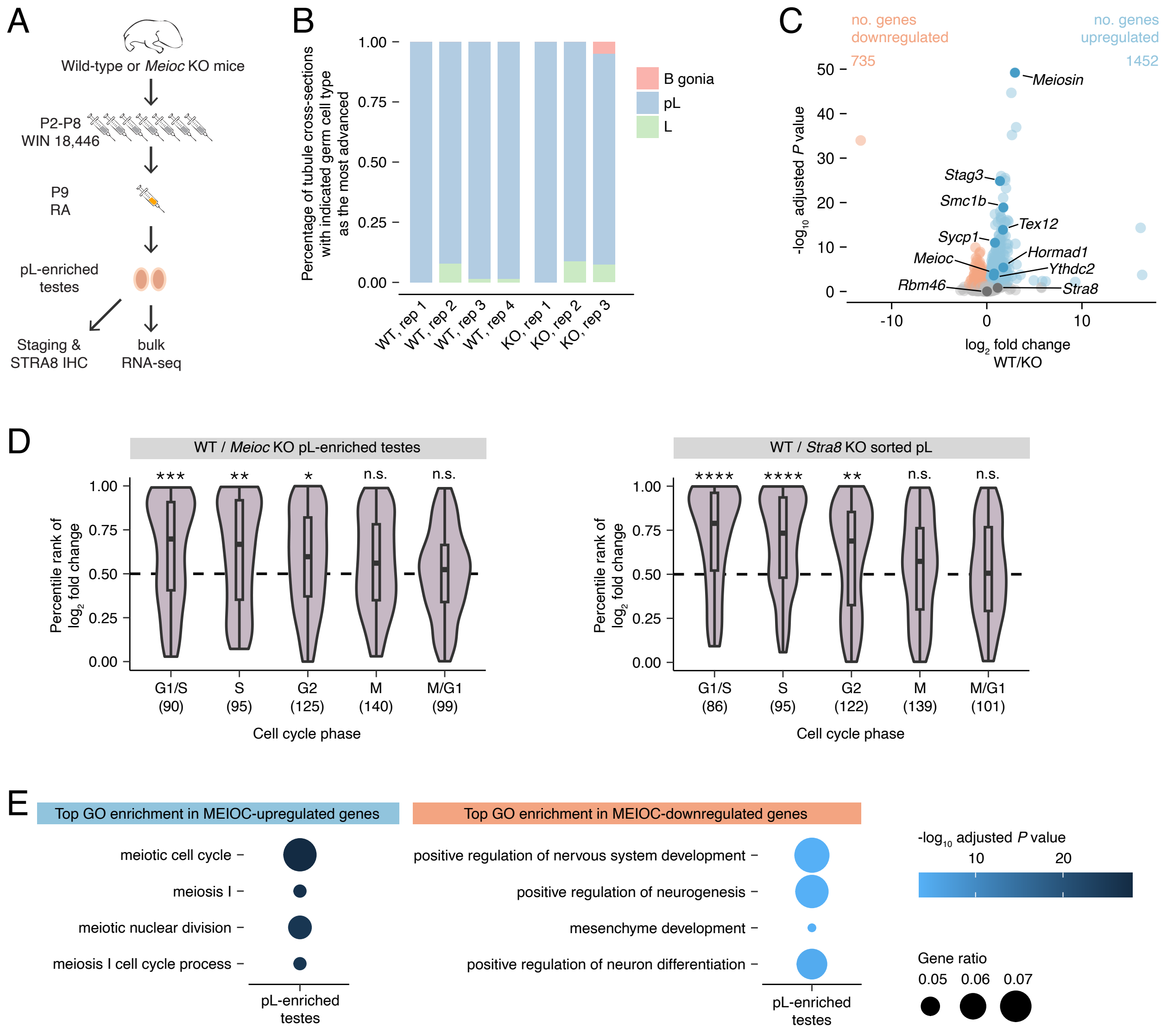
A
Wild-type or Meioc KO mice
P2-P8
WIN 18,446
P9
RA
pL-enriched
testes
Staging &
STRA8 IHC
bulk
RNA-seq
B
Percentage of tubule cross-sections with indicated germ cell type as the most advanced
1.00
0.75
0.50
0.25
0.00
B gonia
pL
L
WT, rep 1
WT, rep 2
WT, rep 3
WT, rep 4
KO, rep 1
KO, rep 2
KO, rep 3
C
no. genes downregulated
735
no. genes upregulated
1452
-log10 adjusted P value
log2 fold change
WT/KO
Meiosin
Stag3
Smc1b
Tex12
Sycp1
Hormad1
Meioc
Ythdc2
Rbm46
Stra8
D
WT / Meioc KO pL-enriched testes
WT / Stra8 KO sorted pL
Percentile rank of log2 fold change
Cell cycle phase
G1/S (90)
S (95)
G2 (125)
M (140)
M/G1 (99)
G1/S (86)
S (95)
G2 (122)
M (139)
M/G1 (101)
*** ** * n.s. n.s.
**** **** ** n.s. n.s.
E
Top GO enrichment in MEIOC-upregulated genes
meiotic cell cycle
meiosis I
meiotic nuclear division
meiosis I cell cycle process
pL-enriched testes
Top GO enrichment in MEIOC-downregulated genes
positive regulation of nervous system development
positive regulation of neurogenesis
mesenchyme development
positive regulation of neuron differentiation
pL-enriched testes
-log10 adjusted P value
10
20
Gene ratio
0.05
0.06
0.07

Figure S4: MEIOC-dependent program defined by bulk RNA-seq analysis of preleptotene-enriched testes.

A: Developmental synchronization of spermatogenesis for bulk RNA-seq analysis. IHC, immunohisto-chemistry; RA, retinoic acid.

B: Enrichment for preleptotene spermatocytes in developmentally synchronized testes.

C: Differential expression of bulk RNA-seq data. Blue marks genes that are upregulated in response to MEIOC ($\log_2$ fold change>0 and adjusted *P*<0.05). Orange marks genes that are downregulated in response to MEIOC ($\log_2$ fold change<0 and adjusted *P*<0.05). *Rbm46* and *Stra8* are highlighted in dark gray; *Meioc*, *Ythdc2*, and *Meiosin* expression are highlighted in dark blue. Other dark blue genes represent those that fall under meiosis-associated GO terms from panel E.

D: Cell cycle analysis of differential expression from bulk RNA-seq data. MEIOC increases abundance of genes associated with G1/S, S, and G2 phases, similar to STRA8. WT and *Stra8* KO bulk RNA-seq data from sorted preleptotenes were reanalyzed from Kojima et al., 2019.

E: Top four GO gene lists enriched in MEIOC-upregulated and -downregulated genes.

*, adjusted *P*<0.05; **, adjusted *P*<0.01; ***, adjusted *P*<0.001; adjusted *P*<0.0001; n.s., not significant.

# Figure S5

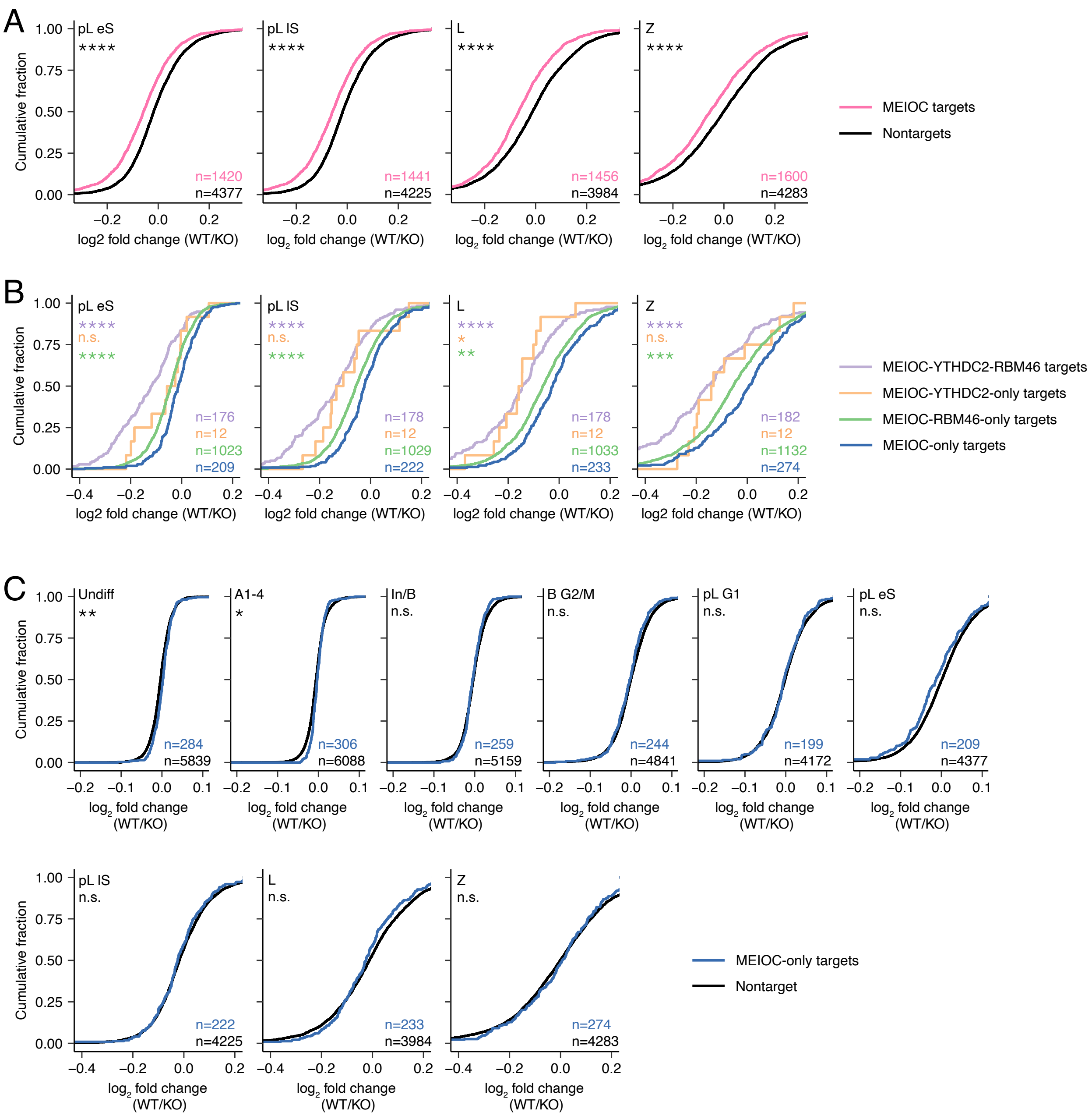
A
B
C
Cumulative fraction
log2 fold change (WT/KO)
pL eS
pL lS
L
Z
Undiff
A1-4
In/B
B G2/M
pL G1
MEIOC targets
Nontargets
MEIOC-YTHDC2-RBM46 targets
MEIOC-YTHDC2-only targets
MEIOC-RBM46-only targets
MEIOC-only targets
Nontarget
n=1420
n=4377
n=1441
n=4225
n=1456
n=3984
n=1600
n=4283
n=176
n=12
n=1023
n=209
n=178
n=1029
n=222
n=1033
n=233
n=182
n=1132
n=274
n=284
n=5839
n=306
n=6088
n=259
n=5159
n=244
n=4841
n=199
n=4172
n.s.

Figure S5: MEIOC destabilizes its targets, based on scRNA-seq analysis.

A: Cumulative fraction of $log_2$ fold changes in transcript abundance in response to MEIOC (WT/ *Meio*c KO), in MEIOC targets compared to nontargets for pL eS, pL lS, L, and Z clusters. Plots for other clusters shown in Figure 2B.

B: Cumulative fraction of $log_2$ fold changes in transcript abundance in response to MEIOC (WT/ *Meioc* KO), in MEIOC-YTHDC2-RBM46 targets, MEIOC-RBM46-only targets, MEOIC-YTHDC2-only targets, and MEIOC-only targets for pL eS, pL lS, L, and Z clusters. Asterisks represent comparison of color-matched target set to MEIOC-only targets. Plots for other clusters shown in Figure 2E.

C: Cumulative fraction of $log_2$ fold changes in transcript abundance in response to MEIOC (WT/ *Meioc* KO), in MEIOC-only targets compared to nontargets for all germ cell clusters.

*, adj. $P<0.05$; **, adj. $P<0.01$; ***, adj. $P<0.001$; ****, adj. $P<0.0001$; n.s., not significant.

# Figure S6

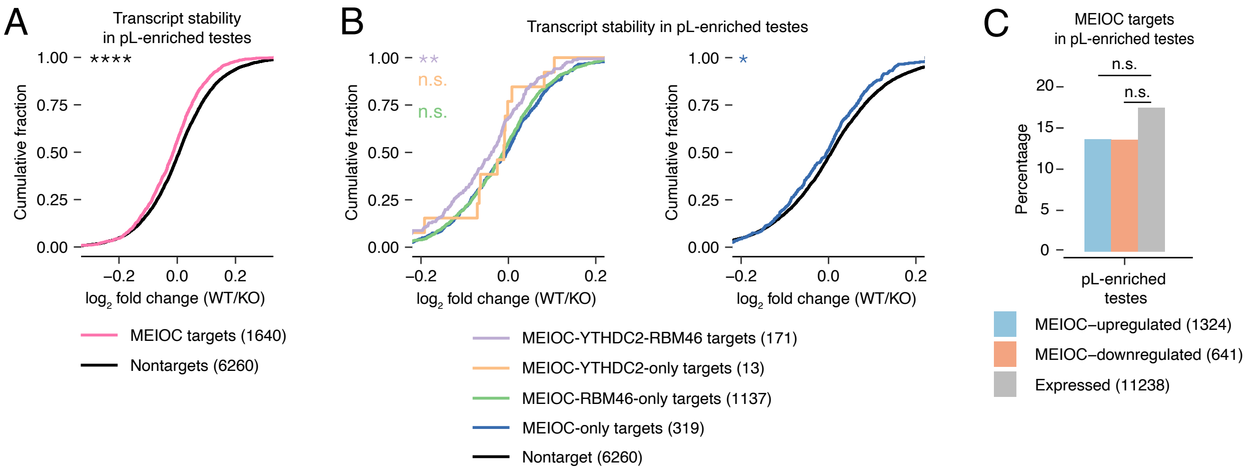


Figure S6: MEIOC destabilizes its targets, based on bulk RNA-seq analysis of preleptotene-enriched testes.

A: Cumulative distribution of $\log_2$ fold change (WT/ *Meioc* KO) in transcript stabliity for MEIOC targets compared to nontargets. Transcript stabiliy was estimated from bulk RNA-seq data of preleptotene-enriched testes.

B: Cumulative distribution of $\log_2$ fold change (WT/ *Meioc* KO) in transcript stabliity for MEIOC-YTHDC2-RBM46 targets, MEIOC-RBM46-only targets, MEIOC-YTHDC2-only targets, and MEIOC-only targets (left), with asterisks representing comparison of color-matched target set to MEIOC-only targets. Cumulative distribution of $\log_2$ fold change (WT/ *Meioc* KO) in transcript stabliity for MEIOC-only targets compared to nontargets (right), with asterisk representing comparison between two groups.

C: Percentage of MEIOC targets among MEIOC-upregulated, MEIOC-downregulated, and expressed genes, defined by bulk RNA-seq analysis of preleptotene-enriched testes.

*, adj. *P*<0.05; **, adj. *P*<0.01; ***, adj. *P*<0.001; ****, adj. *P*<0.0001; n.s., not significant.

# Figure S7

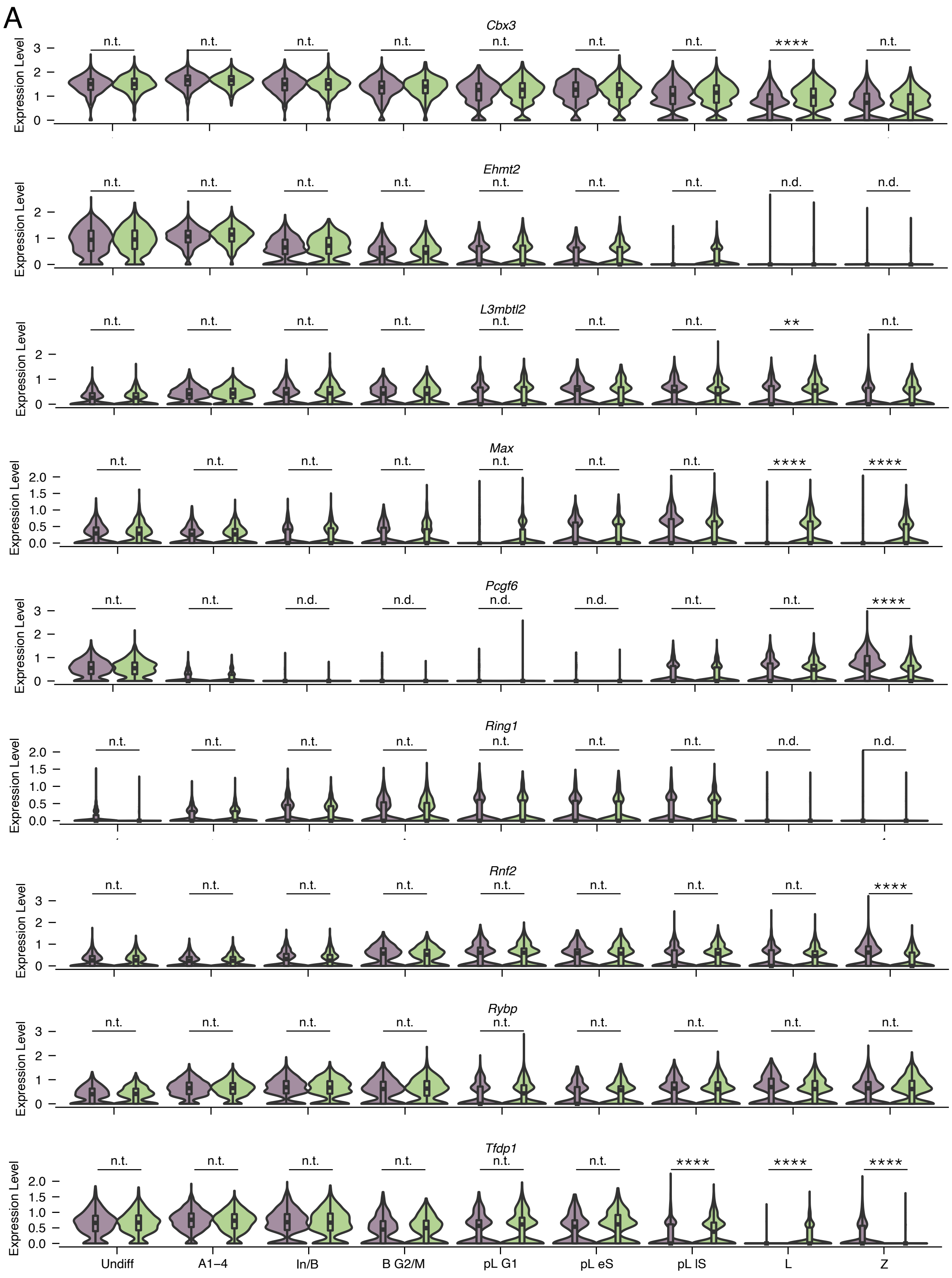
A
Cbx3
Ehmt2
L3mbtl2
Max
Pcgf6
Ring1
Rnf2
Rybp
Tfdp1
Expression Level
n.t.
n.d.
****
**
Undiff
A1–4
In/B
B G2/M
pL G1
pL eS
pL lS
L
Z

Figure S7: Expression of PRC1.6 subunits in wild-type and *Meioc*-null cells from scRNA-seq data of P15 testes.

A: Expression levels of transcripts for PRC1.6 subunits. Clusters marked as “not done” (n.d.) did not meet expression thresholds set for statistical testing.

*, adj. $P$<0.05; **, adj. $P$<0.01; ***, adj. $P$<0.001; ****, adj. $P$<0.0001; n.s., not significant; n.t., not tested (comparison was excluded from statistical testing because $\log_2$ fold change> -0.1 and <0.1); n.d., not detected (transcript expressed in <25% cells in each population being compared).

Figure S8

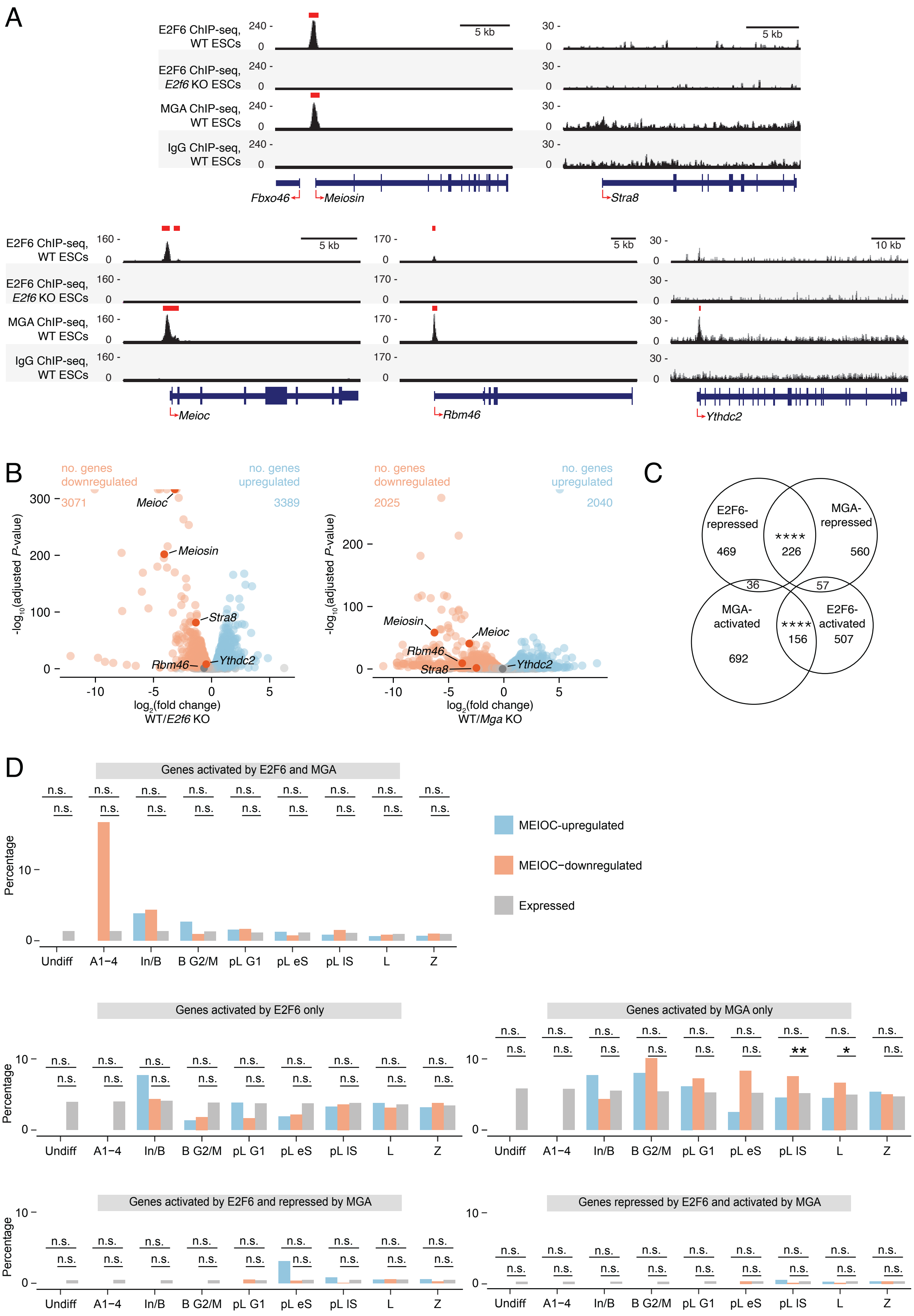
A
E2F6 ChIP-seq, WT ESCs
E2F6 ChIP-seq, E2f6 KO ESCs
MGA ChIP-seq, WT ESCs
IgG ChIP-seq, WT ESCs
5 kb
10 kb
Fbxo46
Meiosin
Stra8
Meioc
Rbm46
Ythdc2
B
no. genes downregulated
no. genes upregulated
3071
3389
2025
2040
-log10(adjusted P-value)
log2(fold change) WT/E2f6 KO
log2(fold change) WT/Mga KO
C
E2F6-repressed
MGA-repressed
MGA-activated
E2F6-activated
469
226
560
36
57
692
156
507
D
Genes activated by E2F6 and MGA
Genes activated by E2F6 only
Genes activated by MGA only
Genes activated by E2F6 and repressed by MGA
Genes repressed by E2F6 and activated by MGA
MEIOC-upregulated
MEIOC−downregulated
Expressed
Percentage
Undiff
A1−4
In/B
B G2/M
pL G1
pL eS
pL lS
L
Z
n.s.

Figure S8: MEIOC-YTHDC2-RBM46's repression of *E2f6* and *Mga* mRNA relieves E2F6- and MGA-mediated transcriptional repression.

A: Input-subtracted E2F6 ChIP-seq signal from wild-type and E2F6 KO ESCs as well as normalized MGA and IgG ChIP-seq signal from wild-type ESCs at the promoters of *Meiosin*, *Stra8*, *Meioc*, *Rbm46*, and *Ythdc2*. Called peaks are marked by a red bar above. Transcriptional start sites are marked by red arrows. E2F6 ChIP-seq data were reanalyzed from Dahlet et al., 2021; MGA ChIP-seq data were reanalyzed from Stielow et al., 2018.

B: *E2f6*-dependent and *Mga*-dependent differential expression program in mouse embryonic stem cells. RNA-seq data were reanalyzed from Dahlet et al., 2021 and Qin et al., 2021. $\text{Log}_2$ fold change was defined as WT/KO. Genes upregulated or downregulated by E2F6 or MGA are shown in blue and orange, respectively. Gray represents genes that do not change expression in response to E2F6 or MGA.

C: Overlap of E2F6-repressed and -activated genes with MGA-repressed and -activated genes, identified from reanalysis of published ChIP-seq and RNA-seq datasets from mouse embryonic stem cells.

D: Percentage of E2F6- and/or MGA-regulated genes among MEIOC-upregulated, MEIOC-downregulated, and expressed genes, as identified via scRNA-seq analysis. Each E2F6/MGA gene set was tested for enrichment among MEIOC-upregulated genes and MEIOC-downregulated genes relative to expressed genes. E2F6-regulated genes and MGA-regulated genes were identified as shown in panel C.

*, adj. $P$<0.05; **, adj. $P$<0.01; ***, adj. $P$<0.001; ****, adj. $P$<0.0001; n.s., not significant.

Figure S9

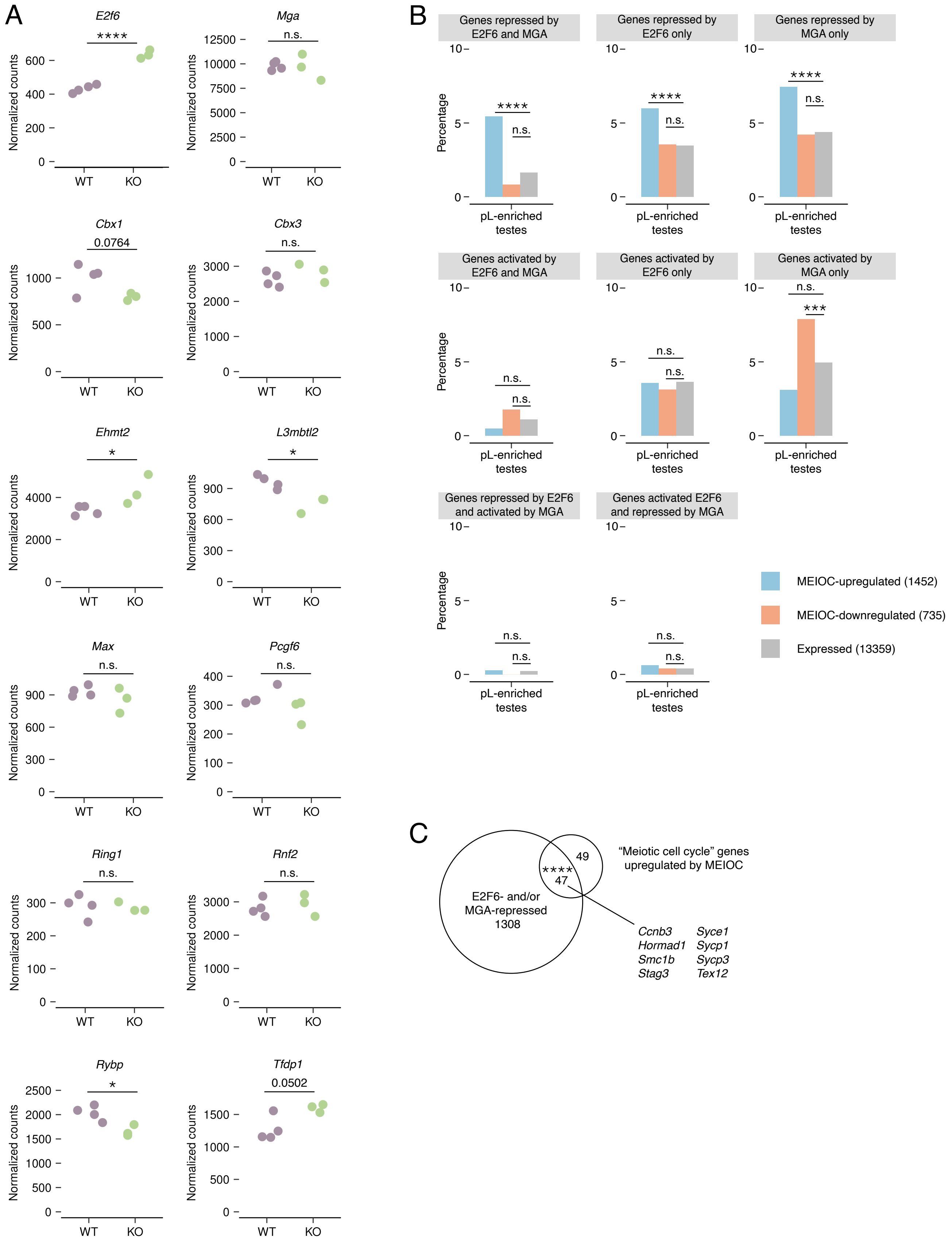
A
E2f6
****
Mga
n.s.
Cbx1
0.0764
Cbx3
n.s.
Ehmt2
*
L3mbtl2
*
Max
n.s.
Pcgf6
n.s.
Ring1
n.s.
Rnf2
n.s.
Rybp
*
Tfdp1
0.0502
Normalized counts
WT
KO
B
Genes repressed by E2F6 and MGA
Genes repressed by E2F6 only
Genes repressed by MGA only
Genes activated by E2F6 and MGA
Genes activated by E2F6 only
Genes activated by MGA only
Genes repressed by E2F6 and activated by MGA
Genes activated E2F6 and repressed by MGA
Percentage
pL-enriched testes
****
***
n.s.
MEIOC-upregulated (1452)
MEIOC-downregulated (735)
Expressed (13359)
C
E2F6- and/or MGA-repressed 1308
****
47
49
"Meiotic cell cycle" genes upregulated by MEIOC
Ccnb3
Hormad1
Smc1b
Stag3
Syce1
Sycp1
Sycp3
Tex12

Figure S9: MEIOC-YTHDC2-RBM46 repression of *E2f6* and *Mga* mRNA relieves E2F6- and MGA-mediated transcriptional repression, based on bulk RNA-seq analysis.

A: Normalized counts in WT vs. *Meioc* KO bulk RNA-seq analysis of preleptotene-enriched testes for *E2f6*, *Mga*, and other subunits of PRC1.6.

B: Percentage of E2F6-repressed and -activated, as well as MGA-repressed and -activated, genes among MEIOC-upregulated, MEIOC-downregulated, and expressed genes from bulk RNA-seq analysis of preleptotene-enriched testes. E2F6 and MGA targets as defined in Figure S8C.

C: Overlap between between E2F6- and/or MGA-repressed genes and the "meiotic cell cycle" genes upregulated by MEIOC in preleptotene-enriched testes.

*, adj. $P<0.05$; **, adj. $P<0.01$; ***, adj. $P<0.001$; ****, adj. $P<0.0001$; n.s., not significant.

Figure S10

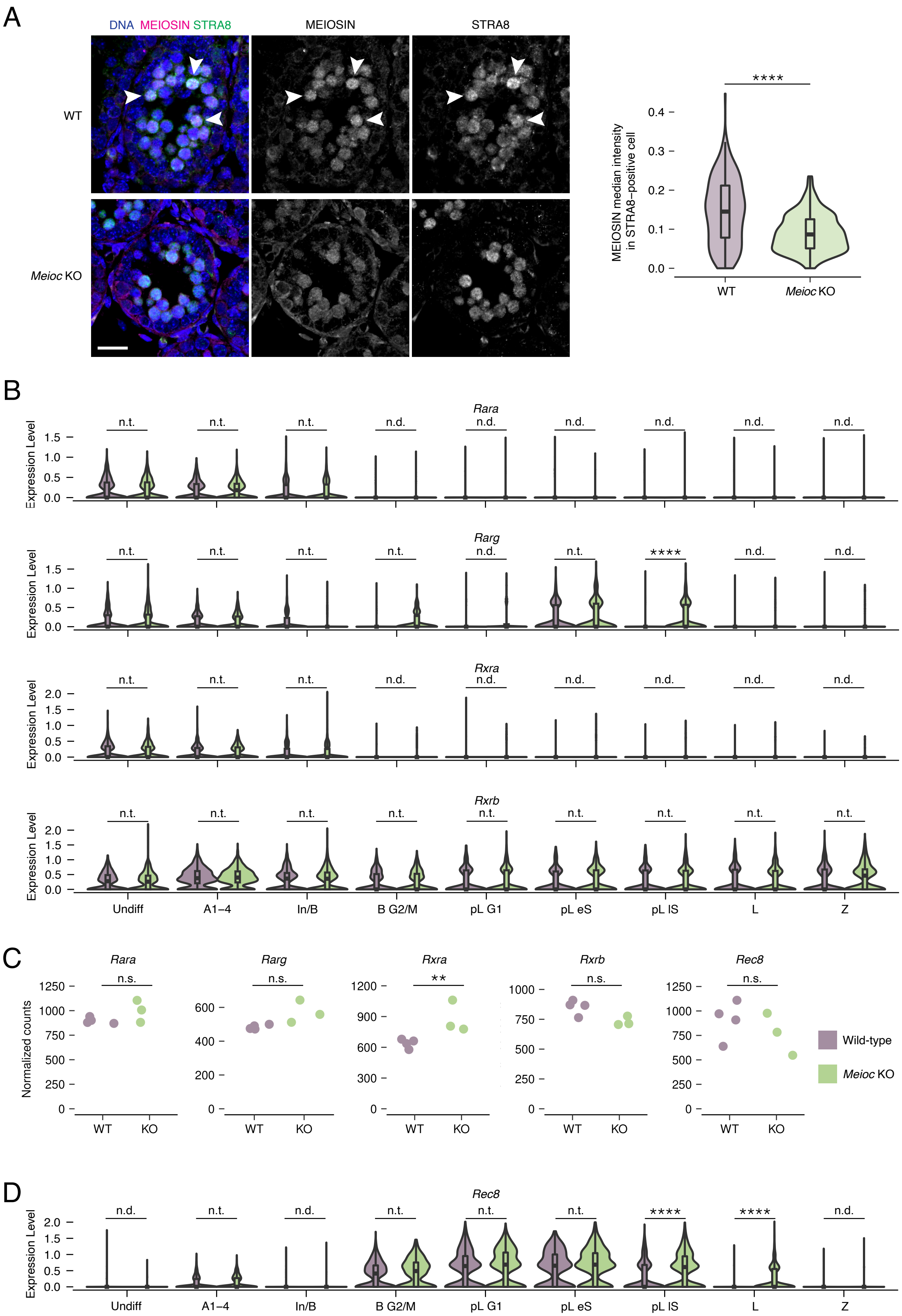

A
DNA MEIOSIN STRA8
MEIOSIN
STRA8
WT
Meioc KO
MEIOSIN median intensity in STRA8–positive cell
****
0.4
0.3
0.2
0.1
0.0
WT
Meioc KO
B
Rara
Rarg
Rxra
Rxrb
Expression Level
n.t.
n.d.
****
Undiff
A1–4
In/B
B G2/M
pL G1
pL eS
pL lS
L
Z
C
Rara
Rarg
Rxra
Rxrb
Rec8
n.s.
**
Normalized counts
WT
KO
Wild-type
Meioc KO
D
Rec8
n.d.
n.t.
****
Expression Level
Undiff
A1–4
In/B
B G2/M
pL G1
pL eS
pL lS
L
Z

Figure S10: MEIOC increases *Meiosin* gene expression in response to retinoic acid.

A: MEIOSIN protein expression in STRA8-positive preleptotene spermatocytes from wild-type and *Meioc* KO P10 testes. Arrowheads represent STRA8-positive spermatocytes with particularly robust MEIOSIN that are absent from *Meioc* KO testes. Graph represents MEIOSIN median intensity from three WT and *Meioc* KO littermate pairs. 75 cells were quantified per testis. Scale bar = 20 um.

B: Expression levels of RAR and RXR genes in wildtype vs. *Meioc*-null cells in all germ cell clusters identified in the scRNA-seq analysis. *Rarb* and *Rxrg* were not detected as expressed. Color legend same as in panel C. Clusters marked as “not done” (n.d.) did not meet expression thresholds set for statistical testing.

C: Normalized counts of RAR and RXR genes as well as *Rec8* in bulk RNA-seq analysis of wildtype vs. *Meioc*-null preleptotene-enriched testes. *Rarb* and *Rxrg* were not detected as expressed.

D: Expression levels of *Rec8* in wildtype vs. *Meioc*-null cells in all germ cell clusters identified in the scRNA-seq analysis. Color legend same as in panel C. Clusters marked as “not done” (n.d.) did not meet expression thresholds set for statistical testing.

*, adj. $P<0.05$; **, adj. $P<0.01$; ***, adj. $P<0.001$; ****, adj. $P<0.0001$; n.s., not significant; n.t., not tested (comparison was excluded from statistical testing because $\log_2$ fold change> -0.1 and <0.1); n.d., not detected (transcript expressed in <25% cells in each population being compared).

# Figure S11

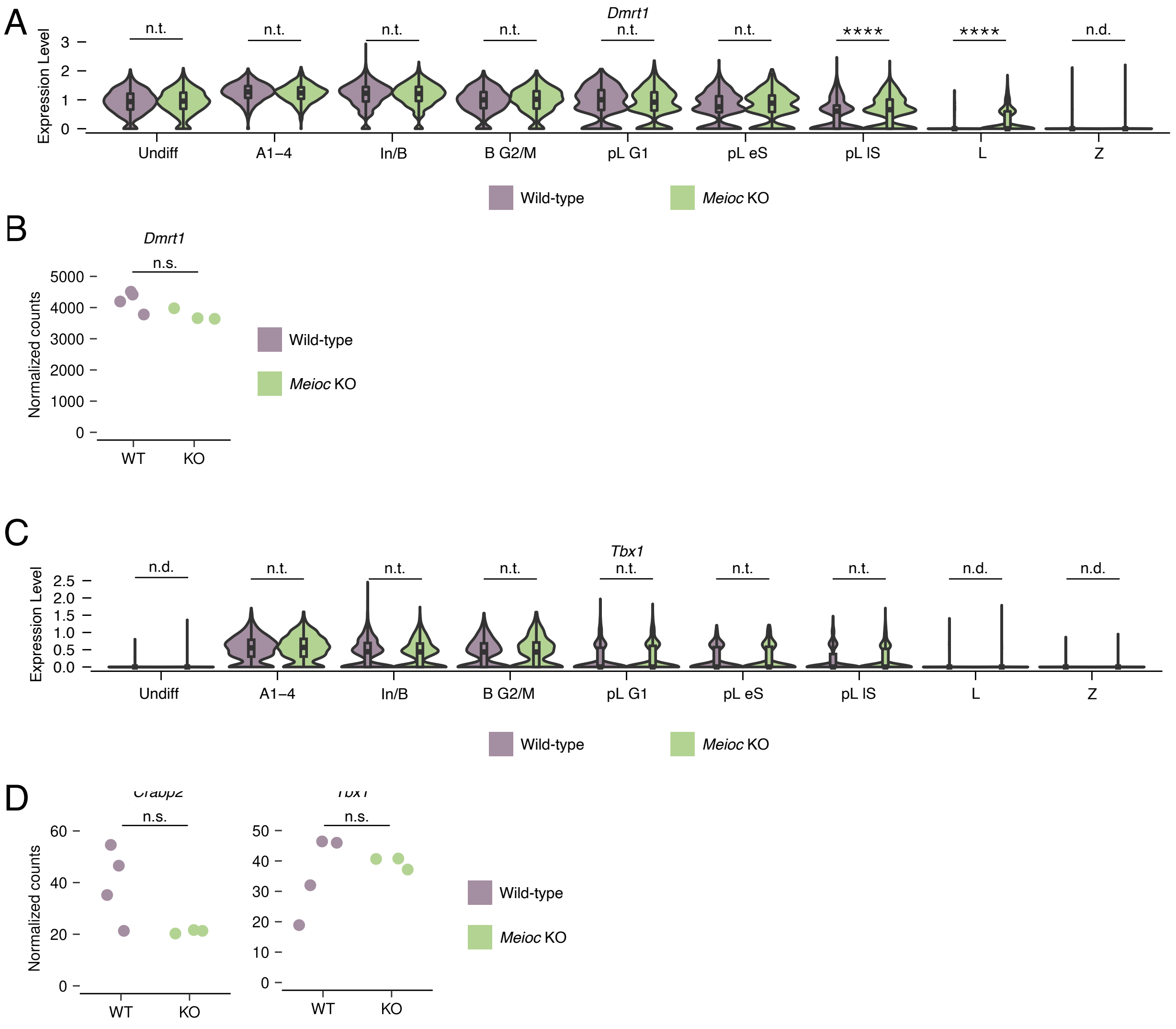


Figure S11: MEIOC does not affect DMRT1 expression or DMRT1-dependent signaling during the mitosis-to-meiosis transition.

A: Expression levels of *Dmrt1* in wildtype vs. *Meioc*-null cells in all germ cell clusters identified in the scRNA-seq analysis. Cluster marked as “not done” (n.d.) did not meet expression thresholds set for statistical testing.

B: Normalized counts of *Dmrt1* in bulk RNA-seq anlaysis of wildtype vs. *Meioc*-null preleptotene-enriched testes.

C: Expression levels of DMRT1-regulated gene *Tbx1* in wildtype vs. *Meioc*-null cells in all germ cell clusters identified in the scRNA-seq analysis. Clusters marked as “not done” (n.d.) did not meet expression thresholds set for statistical testing. *Crabp2* was not detected as expressed.

D: Normalized counts of DMRT1-regulated genes *Crabp2* and *Tbx1* in bulk RNA-seq anlaysis of wildtype vs. *Meioc*-null preleptotene-enriched testes.

*, adj. *P*<0.05; **, adj. *P*<0.01; ***, adj. *P*<0.001; ****, adj. *P*<0.0001; n.s., not significant; n.t., not tested (comparison was excluded from statistical testing because $\log_2$ fold change> -0.1 and <0.1); n.d., not detected (transcript expressed in <25% cells in each population being compared).

# Figure S12

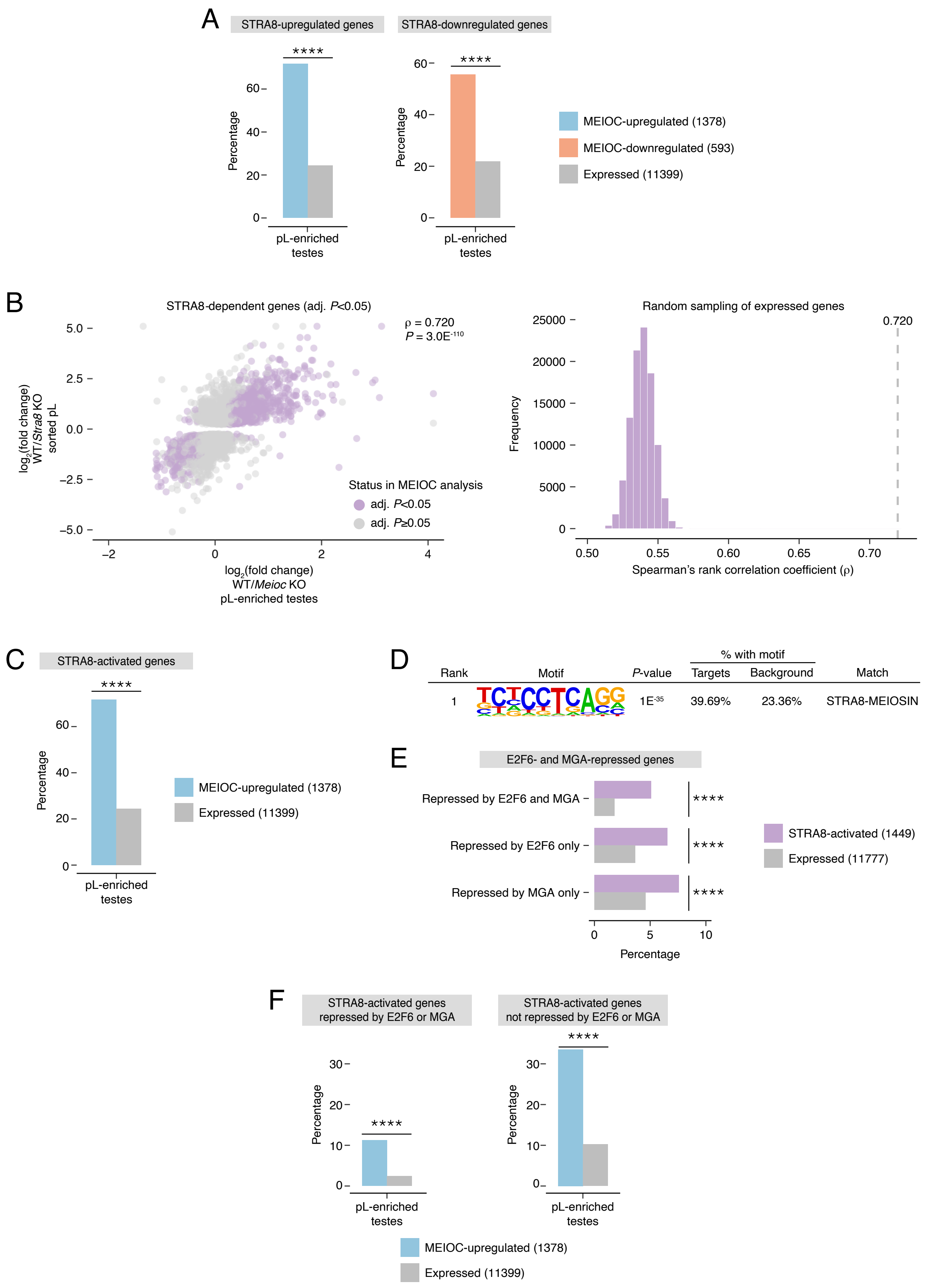
A
STRA8-upregulated genes
STRA8-downregulated genes
Percentage
****
pL-enriched testes
MEIOC-upregulated (1378)
MEIOC-downregulated (593)
Expressed (11399)
B
STRA8-dependent genes (adj. P<0.05)
ρ = 0.720
P = 3.0E-110
log2(fold change) WT/Stra8 KO sorted pL
log2(fold change) WT/Meioc KO pL-enriched testes
Status in MEIOC analysis
adj. P<0.05
adj. P≥0.05
Random sampling of expressed genes
0.720
Frequency
Spearman's rank correlation coefficient (ρ)
C
STRA8-activated genes
Percentage
****
pL-enriched testes
MEIOC-upregulated (1378)
Expressed (11399)
D
Rank
Motif
P-value
% with motif
Targets
Background
Match
1
1E-35
39.69%
23.36%
STRA8-MEIOSIN
E
E2F6- and MGA-repressed genes
Repressed by E2F6 and MGA
Repressed by E2F6 only
Repressed by MGA only
****
Percentage
STRA8-activated (1449)
Expressed (11777)
F
STRA8-activated genes repressed by E2F6 or MGA
STRA8-activated genes not repressed by E2F6 or MGA
Percentage
****
pL-enriched testes
MEIOC-upregulated (1378)
Expressed (11399)

Figure S12: MEIOC's derepression of *Meiosin* gene expression activates the STRA8-MEIOSIN transcriptional program in bulk RNA-seq data of preleptotene-enriched testes.

A: Percentage of STRA8-upregulated and -downregulated genes in MEIOC-upregulated, -downregulated, and expressed genes from bulk RNA-seq analysis of preleptotene-enriched testes. STRA8-upregulated and -downregulated genes were identified via reanalysis of bulk RNA-seq data from wild-type and *Stra8* KO sorted preleptotene spermatocytes from Kojima et al., 2019.

B: Left panel, correlation between MEIOC-dependent changes from bulk RNA-seq analysis of preleptotene-enriched testes and STRA8-dependent changes from bulk RNA-seq analysis of sorted preleptotene spermatocytes. Analysis was limited to genes that were statistically dependent on STRA8 (adjusted $P$<0.05). Right panel, distribution of correlations for gene sets randomly sampled from genes expressed in the two bulk RNA-seq datasets.

C: Percentage of STRA8-activated genes in MEIOC-upregulated, and expressed genes from bulk RNA-seq analysis of preleptotene-enriched testes. STRA8-activated genes were identified as those genes with STRA8-bound promoters (as identified by Kojima et al. (2019) via STRA8-FLAG ChIP-seq in preleptotene-enriched testes) and upregulated by STRA8 (as identified by reanalysis of bulk RNA-seq data from wild-type and *Stra8* KO sorted preleptotene spermatocytes from Kojima et al., 2019).

D. Motif enrichment within promoters of MEIOC-upregulated genes from bulk RNA-seq analysis of preleptotene-enriched testes.

E: Percentage of genes repressed by both E2F6 and MGA; E2F6 only; and MGA only in STRA8-activated and all expressed genes. Genes repressed by E2F6 and MGA were defined in mouse embyronic stem cells as shown in Figure S8C.

F: Percentage of genes that are activated by STRA8 and repressed by E2F6 or MGA; and activated by STRA8 but not repressed by E2F6 or MGA among MEIOC-upregulated and expressed genes from bulk RNA-seq analysis of preleptotene-enriched testes. Genes repressed by E2F6 or MGA are defined as those that are repressed by both E2F6 and MGA; by E2F6 only; or by MGA only, as shown in Figure S8C.

*, adj. $P$<0.05; **, adj. $P$<0.01; ***, adj. $P$<0.001; ****, adj. $P$<0.0001; n.s., not significant.

Figure S13

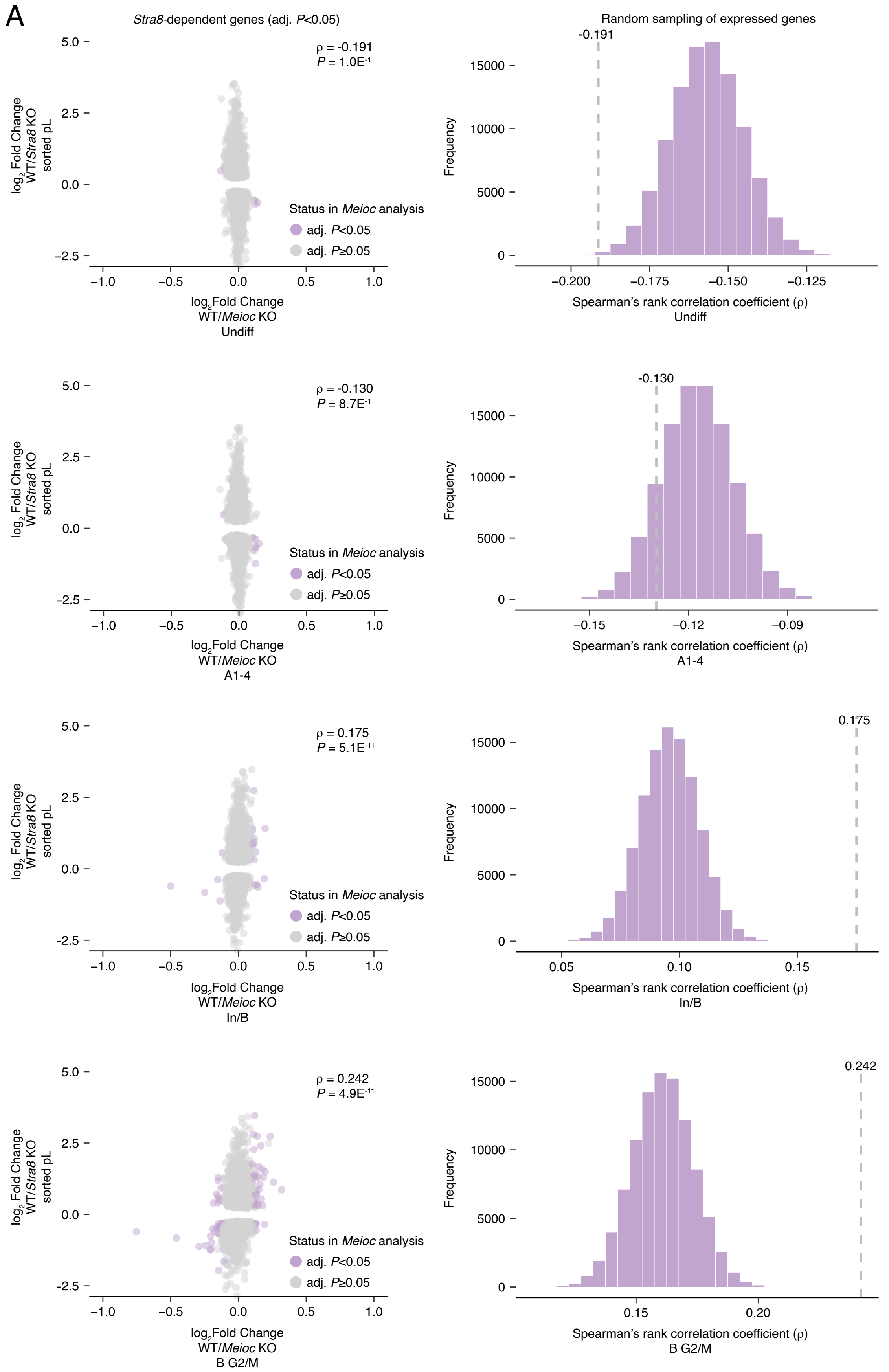
A
Stra8-dependent genes (adj. P<0.05)
Random sampling of expressed genes
ρ = -0.191
P = 1.0E-1
Status in Meioc analysis
adj. P<0.05
adj. P≥0.05
log2 Fold Change WT/Stra8 KO sorted pL
log2Fold Change WT/Meioc KO Undiff
Frequency
-0.191
Spearman's rank correlation coefficient (ρ) Undiff
ρ = -0.130
P = 8.7E-1
log2Fold Change WT/Meioc KO A1-4
-0.130
Spearman's rank correlation coefficient (ρ) A1-4
ρ = 0.175
P = 5.1E-11
log2Fold Change WT/Meioc KO In/B
0.175
Spearman's rank correlation coefficient (ρ) In/B
ρ = 0.242
P = 4.9E-11
log2Fold Change WT/Meioc KO B G2/M
0.242
Spearman's rank correlation coefficient (ρ) B G2/M

Figure S13: Correlation between the MEIOC-dependent program and STRA8-dependent program: A1-4, In/B, and B G2/M clusters.

A: Left panels, correlation between MEIOC-dependent changes from scRNA-seq analysis of Undiff, A1-4, In/B, and B G2/M clusters and STRA8-dependent changes from bulk RNA-seq analysis of sorted preleptotene spermatocytes. Analysis was limited to genes that were statistically dependent on STRA8 (adjusted $P<0.05$). Right panels, distribution of correlations for gene sets randomly sampled from genes expressed in the scRNA-seq Undiff, A1-4, In/B, and B G2/M clusters and bulk RNA-seq sorted preleptotene spermatocytes.

# Figure S14

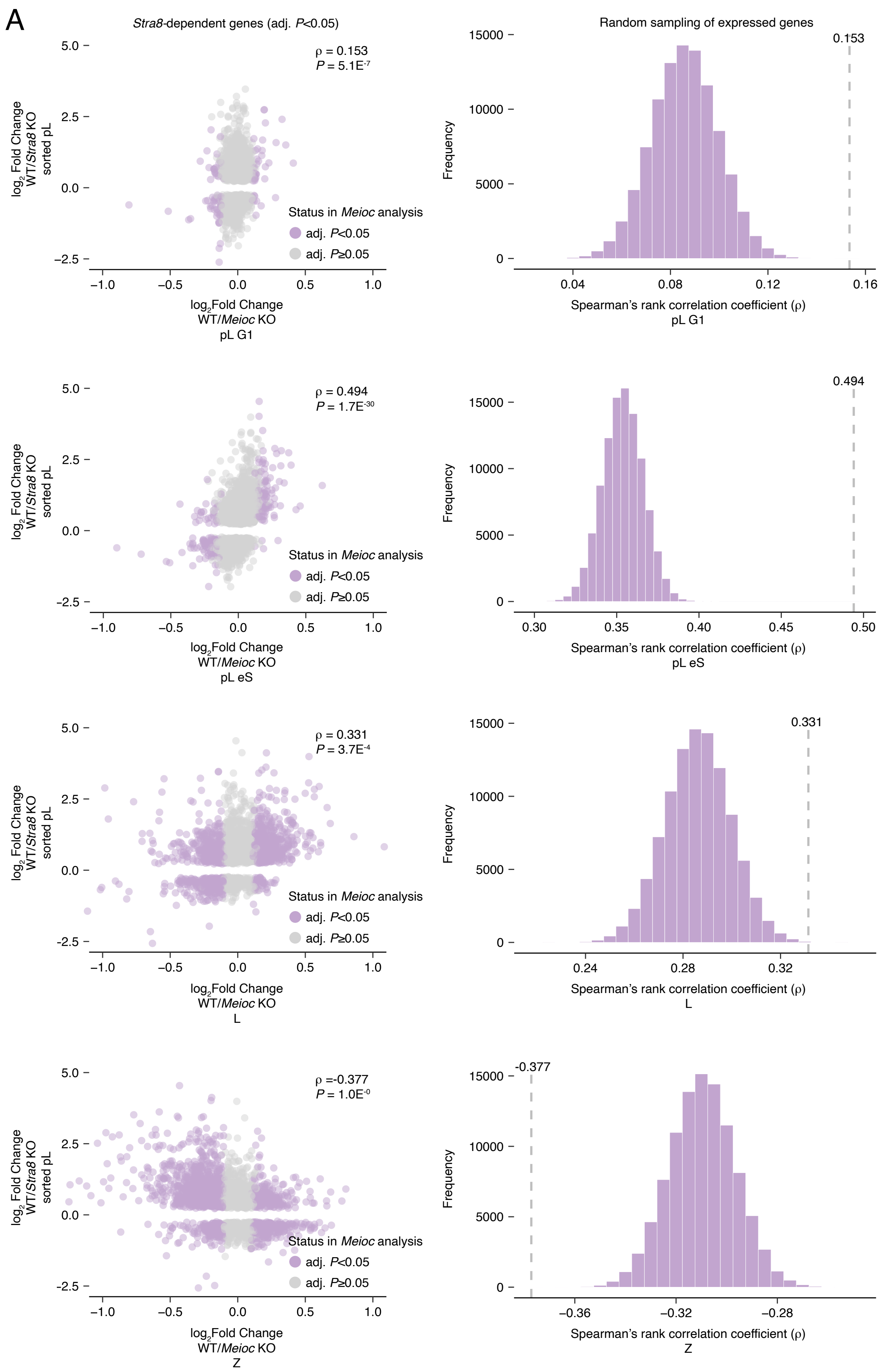
A
Stra8-dependent genes (adj. P<0.05)
Random sampling of expressed genes
ρ = 0.153
P = 5.1E-7
log2 Fold Change WT/Stra8 KO sorted pL
Status in Meioc analysis
adj. P<0.05
adj. P≥0.05
log2Fold Change WT/Meioc KO pL G1
Frequency
0.153
Spearman's rank correlation coefficient (ρ) pL G1
ρ = 0.494
P = 1.7E-30
log2Fold Change WT/Meioc KO pL eS
0.494
Spearman's rank correlation coefficient (ρ) pL eS
ρ = 0.331
P = 3.7E-4
log2Fold Change WT/Meioc KO L
0.331
Spearman's rank correlation coefficient (ρ) L
ρ =-0.377
P = 1.0E-0
log2Fold Change WT/Meioc KO Z
-0.377
Spearman's rank correlation coefficient (ρ) Z

Figure S14: Correlation between the MEIOC-dependent program and STRA8-dependent program: pL G1, pL eS, L, or Z clusters.

A: Left panels, correlation between MEIOC-dependent changes from scRNA-seq analysis of pL G1, pL eS, L, or Z clusters and STRA8-dependent changes from bulk RNA-seq analysis of sorted preleptotene spermatocytes. Analysis was limited to genes that were statistically dependent on STRA8 (adjusted $P<0.05$). Right panels, distribution of correlations for gene sets randomly sampled from genes expressed in the scRNA-seq pL G1, pL eS, L, or Z clusters and bulk RNA-seq sorted preleptotene spermatocytes.
