# Supplementary material for: Post-transcriptional repression of mRNA enhances competence to transit from mitosis to meiosis in mouse spermatogenic cells
Source: bioRxiv. 2023 Sep 22:2023.09.20.557439. Preprint. [Version 1] doi: 10.1101/2023.09.20.557439 (PMC10541148; doi:10.1101/2023.09.20.557439)
Supplement: Supplement 2 [file media-2.pdf]

SUPPLEMENTAL INFORMATION

Supplemental Results

Identification of cell types in scRNA-seq data from P15 testes

We analyzed the P15 postnatal testis from wild-type and *Meioc*-null pups (N=2 per genotype) via 10x Genomics Chromium-based scRNA-seq. Using Seurat for integration (Stuart et al. 2019), we detected the somatic cell types previously identified by 10x scRNA-seq in the postnatal testis (Ernst et al. 2019) (Fig. S1A,B): fetal Leydig cells (*Dlk1*, *Cyp11a1*) (Kaftanovskaya et al. 2015; Ye et al. 2017), peritubular myoid cells (*Acta2*, *Myh11*) (Cool et al. 2008; Chen et al. 2016), vascular endothelium (*Tm4sf1*) (Shih et al. 2009), testicular macrophages (*Cd14*, *Adgre1*, *Itgam*) (Bhushan et al. 2020; Landmann et al. 2000), and Sertoli cells (*Sox9*, *Cldn11*) (Sekido et al. 2004; Mazaud-Guittot et al. 2010). We also identified one somatic cluster that could not be identified based on marker gene expression.

Germ cells, identified via *Dazl* and *Ddx4*, formed the largest cell cluster, as they are the predominant cell type in P15 testes. Using cell type-enriched markers with the UMAP-based cluster relationships from the wild-type cells only, we assigned subclusters of germ cells to the following cell types (Fig. S1A,B): spermatogonial stem cells (*Id4*, *Gfra1*, *Etv5*) (Helsel et al. 2017; Sun et al. 2015; Wu et al. 2011; He et al. 2007), undifferentiated spermatogonia (*Zbtb16*) (Hobbs et al. 2012), type A spermatogonia (*Kit*, *Stra8*, *Ccnd2*) (Endo et al. 2015; Beumer et al. 2000; Yoshinaga et al. 1991; Schrans-Stassen et al. 1999), intermediate/type B spermatogonia (*Kit*) (Yoshinaga et al. 1991; Schrans-Stassen et al. 1999), preleptotene spermatocytes (*Stra8*), leptotene and zygotene spermatocytes (*Meiob*) (Chen et al. 2018; Souquet et al. 2013), and pachytene spermatocytes (*Piwil1*) (Deng and Lin 2002; Chen et al. 2018). We then transferred

these designations to the overlapping *Meioc*-null clusters. While pachytene spermatocytes are not cytologically detected in *Meioc*-null testes, a small number of *Meioc*-null germ cells were assigned to the pachytene cluster, suggesting that they have transcriptomically advanced to an early pachytene-like stage despite cytological defects. We also identified an additional germ cell cluster composed of *Meioc*-null spermatocytes primarily in G2/M phase that likely represent the *Meioc*-null spermatocytes undergoing aberrant metaphase during meiotic prophase I.

One additional germ cell cluster could not be assigned based on markers alone. This cluster had a median number of features that was lower than any other assigned germ cell subpopulation (Fig. S2A); we therefore designated this unassigned germ cell subpopulation a sequencing artifact that persisted despite our quality filter removing cells with fewer than 1000 features (see Materials and Methods); we dropped this unassigned cluster from subsequent analysis.

Identification of discrete preleptotene clusters in G1, early S, and late S phases in scRNA-seq data

As the PCA analysis initially identified four clusters of preleptotene cells, we hypothesized that these clusters corresponded to the cell cycle state of preleptotene cells, which are either in G1 phase or S phase (Kojima et al. 2019; Chen et al. 2018). Using transcriptome-based cell cycle assignments made via Seurat (see Materials and Methods), we discovered that one preleptotene population was enriched in G1 phase cells while the remaining three were enriched in S phase cells (Fig. S1C). We consequently refined the preleptotene clusters as G1, early S, and late S phase (Fig. S1A-C).

To verify G1, early S, and late S phase designations, we sought further confirmation within the preleptotene clusters for gene expression signatures that were independent of the gene:cell cycle phase pairings assessed by Seurat. First, we confirmed that the G1 preleptotene cluster exhibited reduced *Mki67* (Fig. S3A), whose fluctuations during the mitotic cell cycle reach a nadir in G1 phase. Furthermore, in the G1 preleptotene cluster, the top enriched gene was *Rpl39*, and Gene Set Enrichment Analysis (GSEA) revealed enriched expression in genes falling within ribosome-associated Gene Ontology terms (Fig. S3B). This is consistent with prior observations that high levels of ribosome biogenesis facilitates the mitotic G1-to-S phase transition (Derenzini et al. 2005; Gómez-Herreros et al. 2013; Nosrati et al. 2014; Volarević et al. 2000). By contrast, in the early and late S preleptotene clusters, replication-dependent histones were among the top 10 enriched genes in both clusters, while GSEA highlighted enriched expression in genes associated with GO term "DNA recombination" (Fig. S3B). These gene expression patterns corroborate the preleptotene clusters' G1 and S phase designations.

We then sought to identify additional gene expression patterns that distinguish these three preleptotene subpopulations from other germ cells present at P15. *Stra8* appeared as the most enriched transcript in the early and late S preleptotene clusters but was not enriched in the G1 preleptotene cluster relative to all other germ cell clusters (Fig. S3A,B). By contrast, the G1 preleptotene cluster exhibited the second largest fold enrichment in *Rec8* (Fig. S3A,B), a meiosis-specific cohesion gene that is activated by retinoic acid independent of *Stra8* (Soh et al. 2015). *Rec8* was also enriched to a lesser extent in the other preleptotene clusters as well as the G2/M B spermatogonia cluster (Fig. S3A).

Given that STRA8 acts as a heterodimer with MEIOSIN to activate gene expression during meiotic S phase, we also examined *Meiosin* gene expression. *Meiosin* was only robustly

detected, and statistically enriched, in the early S and late S preleptotene clusters as well as the leptotene cluster (Fig. S3A). Therefore, *Meiosin* expression levels can also distinguish G1 from S phase preleptotenes.

In total, these results confirm the G1, early S, and late S phase designation of the preleptotene clusters. In addition, they demonstrate that the transcript abundance of *Stra8* and *Meiosin*, which encode the key transcription factor for meiotic S phase, as well as the meiotic cohesion *Rec8* distinguish preleptotene spermatocytes in G1 phase and S phase from other stages in the P15 testis.

Bulk RNA-seq analysis of preleptotene-enriched testes demonstrates that MEIOC promotes the meiotic G1-to-S phase transition

As testicular germ cells are classically staged via histology, we sought to use histologically-staged samples to independently verify scRNA-seq results showing that *Meioc*-null germ cells diverge from wild-type germ cells in the G1 phase of the preleptotene stage. We developmentally synchronized spermatogenesis to obtain *Meioc*-null and wild-type testes enriched for preleptotene spermatocytes (Fig. S4A). After histologically verifying staging (Fig. S4B), preleptotene-enriched testes were analyzed via bulk RNA-seq for differential expression (Fig. S4C). We then asked whether MEIOC impacts the G1/S phase transition in the preleptotene-enriched testes. Here, we compared percentile ranks for fold changes (WT/*Meioc* KO) for genes enriched at specific cell cycle phase. As a positive control for disrupted G1/S phase transition, we also analyzed a previously generated bulk RNA-seq dataset of preleptotene spermatocytes sorted from wild-type and *Stra8*-null testes on a C57BL/6 background (Kojima et al. 2019). This analysis revealed that both MEIOC and STRA8 increased transcript abundance

for genes associated with G1/S, S, and G2 (Fig. S4D). This bulk RNA-seq analysis confirms the scRNA-seq results that *Meioc*-null germ cells diverge from their wild-type germ cells as early as meiotic G1/S phase transition and further demonstrates that MEIOC promotes germ cells' progression through the meiotic G1/S transition and S phases.

MEIOC shapes the germline transcriptome

Given that MEIOC destabilizes it targets, we asked if this molecular activity was evident in the total number of transcript molecules and expressed genes detected in the wild-type and *Meioc*-null cells. Indeed, beginning in the mitotic A1-4 and In/B clusters, fewer transcript molecules and expressed genes were detected in wild-type cells compared to *Meioc*-null cells (Fig. S2A). Unexpectedly, these differences disappeared in the B G2/M and pL G1 clusters, perhaps because of additional molecular changes that obfuscate MEIOC's activity at the level of total transcript molecules and genes expressed. However, these differences re-emerged more prominently in the pL lS, L, and Z clusters. Furthermore, none of the somatic clusters exhibited a difference between wild-type and *Meioc*-null cells (Fig. S2B), indicating that the differences in the germ cell clusters are biologically meaningful rather than technical artifacts. Therefore, the total numbers of transcript molecules and expressed genes support a role for MEIOC in reducing transcript abundance.

MEIOC does not increase retinoic acid-dependent transcription

Given that retinoic acid signaling transcriptionally activates *Meiosin* (Ishiguro et al. 2020), we considered whether MEIOC may be enhancing retinoic acid-mediated transcription and consequently increasing *Meiosin* gene expression. Retinoic acid signaling is mediated by

nuclear hormone receptors known as retinoic acid receptors (RARs) and retinoid X receptors (RXRs) (reviewed in Endo et al. 2019). We hypothesized that if MEIOC increases retinoic acid-mediated transcriptional activation, it may do so by increasing the transcript abundance of RARs and RXRs. MEIOC did not increase the transcript abundance of any RAR or RXR isoform in the scRNA-seq or bulk RNA-seq datasets (Fig. S10B,C). *Rxra* transcript abundance was decreased by MEIOC in the bulk RNA-seq dataset, which suggests that MEIOC may limit retinoic acid signaling in preleptotene spermatocytes (Fig. S10C). As MEIOC does not interact with *Rxra* mRNA, this regulation is indirect.

Next, we asked whether, like *Meiosin*, other retinoic acid signaling targets in germ cells exhibited increased transcript abundance in response to MEIOC. First, we examined *Stra8*, which is upregulated by retinoic acid when undifferentiated spermatogonia form differentiating spermatogonia (i.e., the A_{al}-A_1 transition) and again during the transition from mitosis to meiosis. We found that the retinoic acid-mediated increase in *Stra8* expression during these two developmental transitions was still evident (Fig. 4A). Furthermore, MEIOC downregulated *Stra8* abundance overall in the scRNA-seq data (Fig. 4A), which again suggests that MEIOC may limit retinoic acid signaling in preleptotene spermatocytes. As *Stra8* mRNA is not directly bound by MEIOC, the molecular basis for this regulation remains uncharacterized. (*Stra8* was not differentially expressed in the bulk RNA-seq analysis of preleptotene-enriched testes, presumably because these data lacked the precise preleptotene staging that was used in the scRNA-seq analysis.) We also examined the transcript abundance of meiotic cohesin *Rec8,* which is transcriptionally activated by retinoic acid independent of *Stra8* during the transition from mitosis to meiosis (Zhang et al. 2021; Soh et al. 2015; Koubova et al. 2014). MEIOC did not increase *Rec8* transcript abundance (Fig. S10C,D). However, MEIOC is required to reduce

Rec8 abundance in late S phase preleptotene and leptotene spermatocytes). In total, these patterns indicate that MEIOC does not activate *Meiosin* gene expression by increasing retinoic acid-mediated transcription.

MEIOC does not regulate DMRT1

We also considered whether *Dmrt1*, whose protein presumably represses *Meiosin* gene expression (Ishiguro et al. 2020), was regulated by MEIOC-YTHDC2-RBM46. We found that *Dmrt1* transcript was not a target of MEIOC, YTHDC2, or RBM46, nor was it differentially expressed in response to MEIOC in the pL G1 or pL eS clusters of the scRNA-seq analysis or in the preleptotene-enriched testes of the bulk RNA-seq analysis (Fig. S11A,B). Furthermore, we failed to detect changes in transcript abundance that would reflect ectopic DMRT1 activity during mitosis-to-meiosis transition in *Meioc*-null germ cells. For example, DMRT1 activity results in elevated expression of *Tbx1* (Matson et al. 2010), which is normally inhibited by retinoic acid signaling (Roberts et al. 2005), but *Tbx1* expression was not affected by MEIOC (Fig. S11C,D). DMRT1 activates expression of *Crabp2* (Matson et al. 2010), whose protein stimulates retinoic acid-dependent transcription (Dong et al. 1999; Budhu and Noy 2002), but *Crabp2* expression was not affected by MEIOC (Fig. S11D). Finally, DMRT1 also directly inhibits *Stra8* expression (Matson et al. 2010), but as noted above, *Stra8* was not activated by MEIOC (Fig. 4A). Therefore, changes in DMRT1 could not account for MEIOC's upregulation of *Meiosin* gene expression.

Supplemental Materials and Methods

Enzymatic digestion of postnatal testis in preparation for 10x Genomics single-cell RNA-seq

One P15 testes per sample was enzymatically dissociated by first shaking vigorously for 30 seconds and incubating at 35˚C for 7 minutes in 900 ul of 0.1% collagenase type I (Worthington LS004196) in Hank's Balanced Salt Solution (HBSS), supplemented with TURBO DNase (Thermo Fisher Scientific AM2238) for a final concentration of 2U/mL. After allowing the sample to sit for 1 minute such that the seminiferous tubules settled to the bottom of the tube, the interstitium was removed by discarding the resulting supernatant, and the remaining sample was incubated at 35˚C for 25 minutes (with gently pipetting every 5 minutes) in 800 ul of 0.05% trypsin and 0.1% collagenase type I in HBSS, supplemented with TURBO DNase for a final concentration of 2U/mL. The cell suspension was then passed through a 40um nylon strainer, and cell suspension was allowed to digest for 35˚C for an additional 20 minutes. The digestion was quenched by adding 200ul fetal bovine serum (FBS) and gently mixing. The cell suspension was again passed through a 40um nylon strainer. The concentration of the resulting cell suspension was assessed using trypan blue on the Countess Cell Counter. Cells were pelleted at 500g for 4 minutes at room temperature, resuspended in 0.05% bovine serum albumin (BSA) in phosphate buffered saline (PBS) for a target concentration of 1000 cells per microliter, and placed on ice. Viability and concentration were re-assessed ~20 minutes prior to loading on the Chromium Controller using trypan blue on a Countess Cell Counter, at which point all samples had a minimum of 90% viability.

Identification of cell type-specific markers and Gene Set Enrichment Analysis in scRNA-seq data

Using wild-type samples only, markers that were depleted or enriched within a cell type were identified via Seurat's *FindAllMarkers* function without a fold change threshold (options: only.pos = FALSE, logfc.threshold = 0, min.pct = 0.25). Features were scaled and centered via Seurat's *ScaleData* function, using percent mitochondrial reads and batch as variables that were regressed out, before generating a heatmap via the *DoHeatmap* function. Gene Set Enrichment Analysis (GSEA) was done using all genes that were statistically tested within each cluster, with P values of 0 replaced with 10^{-300} (i.e., smaller than the smallest non-zero P value) and ranks based on the signed $-\log_{10} P$ value. GSEA was done on Gene Ontology (GO) terms that contained between 100 and 500 genes via clusterProfiler v3.0.4's *gseGO* function (options: OrgDb = "org.Mm.eg.db", ont = "ALL", minGSSize = 100, maxGSSize = 500, pvalueCutoff = 0.05), and the three GO terms exhibiting the greatest enrichment based on Normalized Enrichment Score (NES) were graphed via enrichplot v1.16.2's *gseaplot2* function in R (Yu et al. 2012).

Synchronization of spermatogenesis

Spermatogenesis was synchronized using a protocol originally developed by Hogarth et al. (Hogarth et al. 2013) and modified by Romer et al. (Romer et al. 2018). Briefly, male mice were injected daily subcutaneously from postnatal day (P) 2 to P8 with WIN 18,446 (Santa Cruz Biotechnology) at 0.1mg/gram body weight and on P9 with retinoic acid (RA; MilliporeSigma) at 0.0125 mg/gram body weight. To obtain testes enriched for preleptotene cells, mice were euthanized and testes were collected at 6.75 days after the RA injection. For each pup, a small testis biopsy was collected for histology to confirm proper enrichment of the desired cell type, and the rest of the testes were flash frozen as whole testes or cell sorted. For histological

verification of staging, the testis biopsy was fixed in Bouin's solution and stained for STRA8, as described below.

Motif analysis of bulk RNA-seq data from preleptotene-enriched testes

De novo motif analysis of the promoters (transcriptional start site ±1000bp) of MEIOC-upregulated genes with the promoters of all expressed genes as background was performed via HOMER motif discovery software v.4.11.1 (Heinz et al. 2010) (options: -len 8,10) with the mm10 genome v.6.3.

Comparison to RNA-seq dataset from Stra8*-null preleptotene spermatocytes*

RNA-seq data from *Stra8*-null preleptotene spermatocytes (N=3) and *Stra8*-heterozygote preleptotene spermatocytes expressing high levels of *Stra8* (i.e., phenotypically wild-type controls; N=2), obtained via synchronization and sorting (NCBI GEO GSE115928; Kojima et al. 2019), was quality filtered, pseudoaligned, and analyzed for differential expression as described above. To determine whether genes upregulated by *Meioc* were enriched for genes also upregulated by *Stra8*; whether genes downregulated by *Meioc* were enriched for genes downregulated by *Stra8*; and whether genes upregulated by *Meioc* were enriched for genes directly activated by *Stra8*, these gene lists were statistically compared using a one-tailed hypergeometric test via the *phyper* function with *lower.tail = F* in R. The linear model between the MEIOC-dependent and STRA8-dependent $\log_2$(fold changes) for *Stra8*-dependent genes only (padj<0.05) was calculated using the *lm* function in R. In the graph of this data, the small number of extreme data points that fell outside of graph's axes are displayed as 0.1 outside of the axis limit.

Gene Ontology analysis of scRNA-seq and bulk RNA-seq data

Within each scRNA-seq cluster, MEIOC-upregulated (log_2 fold change > 0.1 and adjusted *P* value < 0.05) and MEIOC-downregulated genes (log_2 fold change < -0.1 and adjusted *P* value < 0.05) were separately tested for enrichment of Gene Ontology Biological Processes gene lists relative to a background of all expressed genes (i.e., genes expressed in at least 25% of wild-type or *Meioc*-null cells within that cluster). For bulk RNA-seq data, MEIOC-upregulated (log_2 fold change > 0 and adjusted *P* value < 0.05) and MEIOC-downregulated genes (log_2 fold change < -0.1 and adjusted *P* value < 0.05) were similarly tested against a background of all expressed genes (i.e., a minimum TPM of 1 in at least 3 of 7 2S preleptotene samples). These analyses were done via R package clusterProfiler v3.0.4 using the *enrichGO* function (options: OrgDb = "org.Mm.eg.db", ont = "BP", pvalueCutoff = 0.05, readable = T, pAdjustMethod = "BH"). For the scRNA-seq data, the average log_2 fold change for select upregulated and downregulated genes that fell under GO terms “meiotic cell cycle” (GO:0051321) and “mitotic cell cycle” (GO:0000278), respectively, were graphed via heatmap to show gene expression changes between wild-type and *Meioc*-null cells across scRNA-seq germ cell clusters.

Cell cycle analysis of bulk RNA-seq and differentially expressed genes from scRNA-seq’s late S phase preleptotene (pL lS), leptotene (L), and zygotene (Z) clusters

A curated list of human cell cycle genes whose expression is associated with specific cell cycle phases was obtained from Supplementary File S1 from Hsiao et al., 2020. This gene set originated from Macosko et al., 2015 and represents a subset of the genes annotated in Whitfield et al., 2002. (Note that this Macosko et al., 2015 gene list was also used for the cell cycle

analysis implemented for scRNA-seq analysis by Seurat.) These human genes were converted to their one-to-one mouse orthologs, resulting in a list of 564 mouse genes.

For analysis of bulk RNA-seq data, percentile ranks were calculated for all expressed genes, using the $\log_2$ fold changes (WT/KO) from the differential expression results with 0 representing the most downregulated genes and 1 representing the most upregulated genes. Genes' percentile ranks for each cell cycle phase was compared to the mean percentile rank for all expressed genes (0.5) via a two-sided Wilcoxon signed rank test, as implemented by the *wilcox.test* function in R.

To assess what cell cycle phase represented by the enrichment for the "mitotic cell cycle" GO term (GO:0000278) in MEIOC-downregulated genes among the pL, L, and Z clusters, the downregulated genes associated with this GO term were extracted and linked to their associated cell cycle phase (if any), as annotated by Hsiao et al., 2020. The percentage of these genes associated with G1/S, S, G2, M, and M/G1 were graphed. Note that some genes are associated with more than one cell cycle phase and are therefore represented more than once in the graph. Genes whose expression was not associated with a specific cell cycle phase were not graphed.

Re-analysis of MEIOC RIP-seq dataset and comparison to YTHDC2 and RBM46 CLIP datasets

MEIOC RIP-seq from P15 testes (NCBI GEO GSE96920; Soh et al. 2017) was reanalyzed. Reads were quality trimmed using the methods described above. For the MEIOC RIP-seq analysis, the additional cutadapt option --cut 3 was used to remove each sequencing read's first three bases that were added during SMARTer Stranded library preparation. Trimmed reads were pseudoaligned using the methods described above, with the option --fr-stranded for strand-specificity. Quantified transcripts were filtered for protein-coding genes, transcript-level

estimated counts and transcripts per million (TPM) values were summed to the gene level, and TPMs were renormalized to transcript-per-million units. Read counts from kallisto were rounded to the nearest integer and then supplied to DESeq2 v1.26.0.Targets for each RIP-seq dataset were defined using a DESeq model as previously described (Soh et al. 2017), using cutoffs of fold change > 1.5 and adjusted *P* value < 0.05. (This fold change cut off was less stringent than our prior published analysis, which used fold change >3 (Soh et al. 2017).) MEIOC targets and nontargets were limited to those genes with a minimum TPM of 1 in at least 2 of 4 wild-type input RNA-seq samples.

RBM46 eCLIP data from P12-P14 testes (NCBI GEO GSE197282) (Qian et al. 2022) was reanalyzed using Read 1 fastq files only. CLIP data was analyzed as previously described (Busch et al. 2020): sequencing reads were mapped to the mouse genome (mm10) with the GENCODE Basic vM15 gene annotation via STAR v2.7.1a (options: --outFilterMismatchNoverReadLmax 0.04 --outFilterMultimapNmax 1 --alignEndsType Extend5pOfRead1 --outFilterMismatchNmax 2 --outSJfilterReads Unique), and peaks were called via PureCLIP v1.3.1 (Krakau et al. 2017) (options: -nt 8 -ld -iv 'chr1;chr2;chr3;') using two iCLIP biological replicates and one input. Peaks were filtered for those that were supported by both eCLIP biological replicates, annotated using the GENCODE Basic vM15 gene annotation, and assigned to a transcript position based on the following hierarchy: 3' UTR exon > 5' UTR exon> coding exon > intron. In total, we identified 19,667 peaks corresponding to 4,912 protein-coding genes, which is similar to the previously reported 24,010 peaks and 4,413 protein-coding genes (Qian et al. 2022).

The overlap between MEIOC targets and YTHDC2 targets; MEIOC targets and RBM46 targets; MEIOC-YTHDC2 targets and RBM46 targets; as well as MEIOC targets and the

MEIOC-dependent program was tested via a one-tailed hypergeometric test using the *phyper* in R.

Identification of transcriptional repressors that are repressed by MEIOC-YTHDC2-RBM46

To identify transcriptional repressors, the "Function" description of the UniProtKB database (release 2020_01; www.uniprot.org) as manually examined for mouse proteins whose transcripts were (i) downregulated by MEIOC in the In/B, B G2M, and/or pL G1 clusters and (ii) targeted by MEIOC-YTHDC2-RBM46. Any proteins that were annotated as repressing transcription or gene expression were considered transcriptional repressors.

Re-analysis of E2F6 and MGA ChIP-seq datasets from ESCs

E2F6 ChIP-seq and input data from wild-type and *E2f6*-knockout mouse ESCs (NCBI GEO GSE149025) (Dahlet et al. 2021) as well as MGA and IgG ChIP-seq from wild-type mouse ESCs (ArrayExpress E-MTAB-6007) (Stielow et al. 2018) were analyzed. Reads were quality trimmed as described above and mapped to the mouse genome (GRCm38/mm10 assembly) via bowtie2 v.2.3.4.1 (Langmead and Salzberg 2012). ChIP-seq peaks were called via mac2 v.2.2.7.1 (Zhang et al. 2008) with a default P value cutoff of 1×10^{-5}. E2F6 ChIP-seq peaks were called using the corresponding input sample, while MGA ChIP-seq peaks were called using the IgG ChIP-seq sample as the control. Peaks were annotated using annotatePeaks.pl from the HOMER motif discovery software v.4.11.1 (Heinz et al. 2010) with the mm10 genome v.6.3, and those overlapping gene promoters (defined as transcriptional start site ±1000bp) were identified. Peaks identified in *E2f6*-knockout cells were considered background signal from the antibody, and peaks associated with a gene promoter in both the wild-type and *E2f6*-knockout

cells were excluded from subsequent analysis, regardless of the extent of peak overlap between the two samples.

Input-subtracted normalized ChIP-seq signal was visualized by generating a bigWig file with the input chromatin reads subtracted from the ChIP sample reads using the bamCompare from deepTools v.3.5.0 (Ramírez et al. 2016) (options: --operation subtract --scaleFactorsMethod None --normalizeUsing RPKM --ignoreDuplicates --smoothLength 100 --binSize 10). For E2F6 ChIP-seq, input samples were used as input; for MGA ChIP-seq, the IgG ChIP-seq sample was used as input. ChIP-seq signal was visualized using the GRCm38/mm10 genome assembly on the UCSC Genome Browser (http://genome.ucsc.edu/).

Input-subtracted ChIP-seq signal at gene promoters was quantified by first removing potential PCR duplicates using rmdup from samtools v.1.11 (Li et al. 2009). The number of reads at the promoter (transcriptional start site ±1000bp) of each protein-coding transcript from the GENCODE Basic vM15 annotation was determined and summarized on a per-gene basis using htseq-count (options: -m union -t transcript -i gene_name) (Anders et al. 2015). For each gene, counts were normalized to reads per million (rpm) by scaling to the number of unduplicated reads, and the normalized input read count was subtracted from the normalized ChIP-seq-read count.

Re-analysis of STRA8 RNA-seq dataset

RNA-seq data from *Stra8*-null preleptotene spermatocytes (N=3) and *Stra8*-heterozygote preleptotene spermatocytes expressing high levels of *Stra8* (i.e., phenotypically wild-type controls; N=2), obtained via synchronization and sorting (NCBI GEO GSE115928; Kojima et al. 2019), was quality filtered, pseudoaligned, and analyzed for differential expression as described

above. STRA8-upregulated genes were defined as $\log_2$ fold change (WT/*Stra8* KO) > 0 and adjusted P<0.05; STRA8-downregualted genes were defined as $\log_2$ fold change (WT/*Stra8* KO) < 0 and adjusted P<0.05. STRA8-activated genes were defined as the STRA8-upregulated genes whose promoters were also bound by STRA8 (as defined by ChIP-seq in testes enriched for preleptotene spermatocytes (Kojima et al. 2019)).

To determine whether genes upregulated by *Meioc* were enriched for genes also upregulated by *Stra8*; whether genes downregulated by *Meioc* were enriched for genes downregulated by *Stra8*; and whether genes upregulated by *Meioc* were enriched for genes directly activated by *Stra8*, these gene lists were statistically compared using a one-tailed hypergeometric test via the *phyper* function with *lower.tail = F* in R. The linear model between the MEIOC-dependent and STRA8-dependent $\log_2$ fold changes for STRA8-dependent genes only (adjusted P<0.05) was calculated using the *lm* function in R. In the graph of this data, the small number of extreme data points that fell outside of graph's axes are displayed as 0.1 outside of the axis limit.

Re-analysis of RNA-seq datasets from wild-type, E2f6*-knockout, and* Mga*-knockout ESCs*

Mouse ESC RNA-seq data from wild-type and *E2f6*-knockout samples (NCBI GEO GSE149025) (Dahlet et al. 2021) as well as from wild-type and *Mga*-knockout samples (NCBI GEO GSE144141) (Qin et al. 2021) were analyzed. Reads were quality trimmed and pseudoaligned as described above, except without the strand-specific pseudoalignment option. Quantified transcripts were filtered for protein-coding genes, transcript-level estimated counts and transcripts per million (TPM) values were summed to the gene level, and TPMs were renormalized to transcript-per-million units. Read counts from kallisto were rounded to the

nearest integer and then supplied to DESeq2 v1.26.0. Within each dataset, differential expression was determined using a DESeq model that included genotype. Genes were filtered for a minimum TPM of 1 in at least two of six samples.

Genes that exhibited E2F6 ChIP-seq peaks in their promoters and were E2F6-upregulated in the RNA-seq dataset ($\log_2$ fold change (WT/*E2f6* KO) > 0, adj. $P < 0.05$) were defined as directly activated by E2F6. Genes that exhibited E2F6 ChIP-seq peaks in their promoters and were E2F6-downregulated in the RNA-seq dataset ($\log_2$ fold change (WT/*E2f6* KO) < 0, adj. $P < 0.05$) were defined as directly repressed by E2F6. MGA-activated and -repressed genes were similarly defined using the MGA ChIP-seq and RNA-seq datasets.

Chromogenic and fluorescent immunostaining

For chromogenic staining of synchronized testes, testis biopsies were fixed in Bouin's solution for 2 hours at room temperature. For fluorescent staining, dissected testes were fixed in 4% (w/v) paraformaldehyde at 4˚C overnight. Fixed tissues were embedded in paraffin, and 6 um sections were generated. Slides were deparaffinized and hydrated using xylene and ethanol solutions, respectively. For antigen retrieval, slides were boiled in citrate buffer (10 mM sodium citrate, 0.05% Tween 20, pH 6.0) for 10 min.

For chromogenic staining, tissue sections were stained with anti-STRA8 (Abcam ab49405; 1:500 dilution) using ImmPRESS HRP anti-Rabbit Detection Kit (Vector Laboratories MP-7401–50) and ImmPACT DAB Peroxidase Substrate (Vector Laboratories SK-4105). Sections were then washed in PBS, counterstained with hematoxylin, and coverslipped with Permount Mounting Medium.

For fluorescent staining, tissue sections were incubated in blocking solution (10% normal donkey serum in PBS) for 1 hour at room temperature and then with primary antibodies diluted in blocking solution overnight at 4˚C. The following primary antibodies were used: anti-MEIOSIN (guinea pig polyclonal from Ishiguro et al., 2020, 1:100 dilution), and anti-STRA8 (Abcam ab49405, 1:200 dilution). Sections were washed in PBS before incubating with fluorophore-conjugated secondary antibodies diluted at 1:250 in blocking solution for 1 hour at room temperature. The following secondary antibodies were used: donkey anti-guinea pig DyLight 647 (Jackson ImmunoResearch Laboratories 706-605-148) and donkey anti-rabbit AlexaFluor 488 (Jackson ImmunoResearch Laboratories 711-545-152). Sections were washed in PBS, counterstained with DAPI, coverslipped with ProLong Gold Antifade reagent (Thermo Fisher Scientific), and imaged via confocal microscopy (Zeiss LSM 700).

References

Anders S, Pyl PT, Huber W. 2015. HTSeq--a Python framework to work with high-throughput sequencing data. *Bioinforma Oxf Engl* **31**: 166–169.

Beumer TL, Roepers-Gajadien HL, Gademan IS, Kal HB, de Rooij DG. 2000. Involvement of the D-type cyclins in germ cell proliferation and differentiation in the mouse. *Biol Reprod* **63**: 1893–8.

Bhushan S, Theas MS, Guazzone VA, Jacobo P, Wang M, Fijak M, Meinhardt A, Lustig L. 2020. Immune Cell Subtypes and Their Function in the Testis. *Front Immunol* **11**: 2558.

Budhu AS, Noy N. 2002. Direct channeling of retinoic acid between cellular retinoic acid-binding protein II and retinoic acid receptor sensitizes mammary carcinoma cells to retinoic acid-induced growth arrest. *Mol Cell Biol* **22**: 2632–2641.

Busch A, Brüggemann M, Ebersberger S, Zarnack K. 2020. iCLIP data analysis: A complete pipeline from sequencing reads to RBP binding sites. *Methods* **178**: 49–62.

Chen L-Y, Willis WD, Eddy EM. 2016. Targeting the Gdnf Gene in peritubular myoid cells disrupts undifferentiated spermatogonial cell development. *Proc Natl Acad Sci U S A* **113**: 1829–34.

Chen Y, Zheng Y, Gao Y, Lin Z, Yang S, Wang T, Wang Q, Xie N, Hua R, Liu M, et al. 2018. Single-cell RNA-seq uncovers dynamic processes and critical regulators in mouse spermatogenesis. *Cell Res* **28**: 879–896.

Cool J, Carmona FD, Szucsik JC, Capel B. 2008. Peritubular myoid cells are not the migrating population required for testis cord formation in the XY gonad. *Sex Dev Genet Mol Biol Evol Endocrinol Embryol Pathol Sex Determ Differ* **2**: 128–33.

Dahlet T, Truss M, Frede U, Al Adhami H, Bardet AF, Dumas M, Vallet J, Chicher J, Hammann P, Kottnik S, et al. 2021. E2F6 initiates stable epigenetic silencing of germline genes during embryonic development. *Nat Commun* **12**: 3582.

Deng W, Lin H. 2002. miwi, a murine homolog of piwi, encodes a cytoplasmic protein essential for spermatogenesis. *Dev Cell* **2**: 819–30.

Derenzini M, Montanaro L, Chillà A, Tosti E, Vici M, Barbieri S, Govoni M, Mazzini G, Treré D. 2005. Key role of the achievement of an appropriate ribosomal RNA complement for G1-S phase transition in H4-II-E-C3 rat hepatoma cells: Ribosome biogenesis and cell cycle progression. *J Cell Physiol* **202**: 483–491.

Dong D, Ruuska SE, Levinthal DJ, Noy N. 1999. Distinct roles for cellular retinoic acid-binding proteins I and II in regulating signaling by retinoic acid. *J Biol Chem* **274**: 23695–23698.

Endo T, Mikedis MM, Nicholls PK, Page DC, de Rooij DG. 2019. Retinoic acid and germ cell development in the ovary and testis. *Biomolecules* **9**.

Endo T, Romer KA, Anderson EL, Baltus AE, de Rooij DG, Page DC. 2015. Periodic retinoic acid-STRA8 signaling intersects with periodic germ-cell competencies to regulate spermatogenesis. *Proc Natl Acad Sci U S A* **112**: E2347-56.

Ernst C, Eling N, Martinez-Jimenez CP, Marioni JC, Odom DT. 2019. Staged developmental mapping and X chromosome transcriptional dynamics during mouse spermatogenesis. *Nat Commun* **10**: 1251.

Gómez-Herreros F, Rodríguez-Galán O, Morillo-Huesca M, Maya D, Arista-Romero M, de la Cruz J, Chávez S, Muñoz-Centeno MC. 2013. Balanced Production of Ribosome Components Is Required for Proper G1/S Transition in Saccharomyces cerevisiae. *J Biol Chem* **288**: 31689–31700.

He Z, Jiang J, Hofmann M-C, Dym M. 2007. Gfra1 silencing in mouse spermatogonial stem cells results in their differentiation via the inactivation of RET tyrosine kinase. *Biol Reprod* **77**: 723–33.

Heinz S, Benner C, Spann N, Bertolino E, Lin YC, Laslo P, Cheng JX, Murre C, Singh H, Glass CK. 2010. Simple Combinations of Lineage-Determining Transcription Factors Prime cis-Regulatory Elements Required for Macrophage and B Cell Identities. *Mol Cell* **38**: 576–589.

Helsel AR, Yang Q-E, Oatley MJ, Lord T, Sablitzky F, Oatley JM. 2017. ID4 levels dictate the stem cell state in mouse spermatogonia. *Development* **144**: 624–634.

Hobbs RM, Fagoonee S, Papa A, Webster K, Altruda F, Nishinakamura R, Chai L, Pandolfi PP. 2012. Functional Antagonism between Sall4 and Plzf Defines Germline Progenitors. *Cell Stem Cell* **10**: 284–298.

Hogarth CA, Evanoff R, Mitchell D, Kent T, Small C, Amory JK, Griswold MD. 2013. Turning a spermatogenic wave into a tsunami: synchronizing murine spermatogenesis using WIN 18,446. *Biol Reprod* **88**: 1–9.

Hsiao CJ, Tung P, Blischak JD, Burnett JE, Barr KA, Dey KK, Stephens M, Gilad Y. 2020. Characterizing and inferring quantitative cell cycle phase in single-cell RNA-seq data analysis. *Genome Res* **30**: 611–621.

Ishiguro K ichiro, Matsuura K, Tani N, Takeda N, Usuki S, Yamane M, Sugimoto M, Fujimura S, Hosokawa M, Chuma S, et al. 2020. MEIOSIN Directs the Switch from Mitosis to Meiosis in Mammalian Germ Cells. *Dev Cell* **52**: 429-445.e10.

Kaftanovskaya EM, Lopez C, Ferguson L, Myhr C, Agoulnik AI. 2015. Genetic ablation of androgen receptor signaling in fetal Leydig cell lineage affects Leydig cell functions in adult testis. *FASEB J Off Publ Fed Am Soc Exp Biol* **29**: 2327–37.

Kojima ML, de Rooij DG, Page DC. 2019. Amplification of a broad transcriptional program by a common factor triggers the meiotic cell cycle in mice. *eLife* **8**. https://elifesciences.org/articles/43738 (Accessed April 13, 2020).

Koubova J, Hu Y-C, Bhattacharyya T, Soh YQS, Gill ME, Goodheart ML, Hogarth C a, Griswold MD, Page DC. 2014. Retinoic Acid Activates Two Pathways Required for Meiosis in Mice. *PLoS Genet* **10**: e1004541.

Krakau S, Richard H, Marsico A. 2017. PureCLIP: capturing target-specific protein–RNA interaction footprints from single-nucleotide CLIP-seq data. *Genome Biol* **18**: 240.

Landmann R, Müller B, Zimmerli W. 2000. CD14, new aspects of ligand and signal diversity. *Microbes Infect* **2**: 295–304.

Langmead B, Salzberg SL. 2012. Fast gapped-read alignment with Bowtie 2. *Nat Methods* **9**: 357–359.

Li H, Handsaker B, Wysoker A, Fennell T, Ruan J, Homer N, Marth G, Abecasis G, Durbin R, 1000 Genome Project Data Processing Subgroup. 2009. The Sequence Alignment/Map format and SAMtools. *Bioinforma Oxf Engl* **25**: 2078–2079.

Macosko EZ, Basu A, Satija R, Nemesh J, Shekhar K, Goldman M, Tirosh I, Bialas AR, Kamitaki N, Martersteck EM, et al. 2015. Highly Parallel Genome-wide Expression Profiling of Individual Cells Using Nanoliter Droplets. *Cell* **161**: 1202–1214.

Matson CK, Murphy MW, Griswold MD, Yoshida S, Bardwell VJ, Zarkower D. 2010. The Mammalian Doublesex Homolog DMRT1 Is a Transcriptional Gatekeeper that Controls the Mitosis versus Meiosis Decision in Male Germ Cells. *Dev Cell* **19**: 612–624.

Mazaud-Guittot S, Meugnier E, Pesenti S, Wu X, Vidal H, Gow A, Le Magueresse-Battistoni B. 2010. Claudin 11 deficiency in mice results in loss of the Sertoli cell epithelial phenotype in the testis. *Biol Reprod* **82**: 202–13.

Nosrati N, Kapoor NR, Kumar V. 2014. Combinatorial action of transcription factors orchestrates cell cycle-dependent expression of the ribosomal protein genes and ribosome biogenesis. *FEBS J* **281**: 2339–2352.

Qian B, Li Y, Yan R, Han S, Bu Z, Gong J, Zheng B, Yuan Z, Ren S, He Q, et al. 2022. RNA binding protein RBM46 regulates mitotic-to-meiotic transition in spermatogenesis. *Sci Adv* **8**: eabq2945.

Qin J, Wang C, Zhu Y, Su T, Dong L, Huang Y, Hao K. 2021. Mga safeguards embryonic stem cells from acquiring extraembryonic endoderm fates. *Sci Adv* **7**: eabe5689.

Ramírez F, Ryan DP, Grüning B, Bhardwaj V, Kilpert F, Richter AS, Heyne S, Dündar F, Manke T. 2016. deepTools2: a next generation web server for deep-sequencing data analysis. *Nucleic Acids Res* **44**: W160-165.

Roberts C, Ivins SM, James CT, Scambler PJ. 2005. Retinoic acid down-regulates Tbx1 expression in vivo and in vitro. *Dev Dyn Off Publ Am Assoc Anat* **232**: 928–938.

Romer KA, de Rooij DG, Kojima ML, Page DC. 2018. Isolating mitotic and meiotic germ cells from male mice by developmental synchronization, staging, and sorting. *Dev Biol* **443**: 19–34.

Schrans-Stassen BH, van de Kant HJ, de Rooij DG, van Pelt AM. 1999. Differential expression of c-kit in mouse undifferentiated and differentiating type A spermatogonia. *Endocrinology* **140**: 5894–900.

Sekido R, Bar I, Narváez V, Penny G, Lovell-Badge R. 2004. SOX9 is up-regulated by the transient expression of SRY specifically in Sertoli cell precursors. *Dev Biol* **274**: 271–9.

Shih S-C, Zukauskas A, Li D, Liu G, Ang L-H, Nagy JA, Brown LF, Dvorak HF. 2009. The L6 protein TM4SF1 is critical for endothelial cell function and tumor angiogenesis. *Cancer Res* **69**: 3272–7.

Soh YQS, Junker JP, Gill ME, Mueller JL, van Oudenaarden A, Page DC. 2015. A Gene Regulatory Program for Meiotic Prophase in the Fetal Ovary. *PLoS Genet* **11**: e1005531.

Soh YQS, Mikedis MM, Kojima M, Godfrey AK, de Rooij DG, Page DC. 2017. Meioc maintains an extended meiotic prophase I in mice ed. P.E. Cohen. *PLOS Genet* **13**: e1006704.

Souquet B, Abby E, Hervé R, Finsterbusch F, Tourpin S, Le Bouffant R, Duquenne C, Messiaen S, Martini E, Bernardino-Sgherri J, et al. 2013. MEIOB Targets Single-Strand DNA and Is Necessary for Meiotic Recombination ed. M. Grelon. *PLoS Genet* **9**: e1003784.

Stielow B, Finkernagel F, Stiewe T, Nist A, Suske G. 2018. MGA, L3MBTL2 and E2F6 determine genomic binding of the non-canonical Polycomb repressive complex PRC1.6 ed. L. Di Croce. *PLOS Genet* **14**: e1007193.

Stuart T, Butler A, Hoffman P, Hafemeister C, Papalexi E, Mauck WM, Hao Y, Stoeckius M, Smibert P, Satija R. 2019. Comprehensive Integration of Single-Cell Data. *Cell* **177**: 1888-1902.e21.

Sun F, Xu Q, Zhao D, Degui Chen C. 2015. Id4 Marks Spermatogonial Stem Cells in the Mouse Testis. *Sci Rep* **5**: 17594.

Volarević S, Stewart MJ, Ledermann B, Zilberman F, Terracciano L, Montini E, Grompe M, Kozma SC, Thomas G. 2000. Proliferation, But Not Growth, Blocked by Conditional Deletion of 40 *S* Ribosomal Protein S6. *Science* **288**: 2045–2047.

Whitfield ML, Sherlock G, Saldanha AJ, Murray JI, Ball CA, Alexander KE, Matese JC, Perou CM, Hurt MM, Brown PO, et al. 2002. Identification of genes periodically expressed in the human cell cycle and their expression in tumors. *Mol Biol Cell* **13**: 1977–2000.

Wu X, Goodyear SM, Tobias JW, Avarbock MR, Brinster RL. 2011. Spermatogonial stem cell self-renewal requires ETV5-mediated downstream activation of Brachyury in mice. *Biol Reprod* **85**: 1114–23.

Ye L, Li X, Li L, Chen H, Ge R-S. 2017. Insights into the Development of the Adult Leydig Cell Lineage from Stem Leydig Cells. *Front Physiol* **8**: 430.

Yoshinaga K, Nishikawa S, Ogawa M, Hayashi S, Kunisada T, Fujimoto T, Nishikawa S. 1991. Role of c-kit in mouse spermatogenesis: identification of spermatogonia as a specific site of c-kit expression and function. *Development* **113**.

Yu G, Wang L-G, Han Y, He Q-Y. 2012. clusterProfiler: an R Package for Comparing Biological Themes Among Gene Clusters. *OMICS J Integr Biol* **16**: 284–287.

Zhang X, Gunewardena S, Wang N. 2021. Nutrient restriction synergizes with retinoic acid to induce mammalian meiotic initiation in vitro. *Nat Commun* **12**: 1758.

Zhang Y, Liu T, Meyer CA, Eeckhoute J, Johnson DS, Bernstein BE, Nusbaum C, Myers RM, Brown M, Li W, et al. 2008. Model-based analysis of ChIP-Seq (MACS). *Genome Biol* **9**: R137.
